# An empirical comparison of sleep-specific versus generic quality of life instruments among Australians with sleep disorders

**DOI:** 10.1007/s11136-024-03686-0

**Published:** 2024-06-24

**Authors:** Taylor-Jade Woods, Billingsley Kaambwa

**Affiliations:** Health Economics Unit, College of Medicine and Public Health, Flinders University, Bedford Park, South Australia Australia

**Keywords:** Quality of life, Sleep health, Sleep disorder, Preference-based instruments, Economic evaluation

## Abstract

**Purpose:**

In Australian adults diagnosed with a sleep disorder(s), this cross-sectional study compares the empirical relationships between two generic QoL instruments, the EuroQoL 5-dimension 5-level (EQ-5D-5L) and ICEpop CAPability measure for Adults (ICECAP-A), and three sleep-specific metrics, the Epworth Sleepiness Scale (ESS), 10-item Functional Outcomes of Sleep Questionnaire (FOSQ-10), and Pittsburgh Sleep Quality Index (PSQI).

**Methods:**

Convergent and divergent validity between item/dimension scores was examined using Kendall’s Tau-B correlation, with correlations below 0.30 considered weak, between 0.30 and 0.50 moderate and those above 0.50 strong (indicating that instruments were measuring similar constructs). Exploratory factor analysis (EFA) was conducted to identify shared underlying constructs.

**Results:**

A total of 1509 participants (aged 18–86 years) were included in the analysis. Convergent validity between dimensions/items of different instruments was weak to moderate. A 5-factor EFA solution, representing ‘daytime dysfunction’, ‘fatigue’, ‘wellbeing’, ‘physical health’, and ‘perceived sleep quality’, was simplest with close fit and fewest cross-loadings. Each instrument’s dimensions/items primarily loaded onto their own factor, except for the EQ-5D-5L and PSQI. Nearly two-thirds of salient loadings were of excellent magnitude (0.72 to 0.91).

**Conclusion:**

Moderate overlap between the constructs assessed by generic and sleep-specific instruments indicates that neither can fully capture the complexity of QoL alone in general disordered sleep populations. Therefore, both are required within economic evaluations. A combination of the EQ-5D-5L and, depending on context, ESS or PSQI offers the broadest measurement of QoL in evaluating sleep health interventions.

**Supplementary Information:**

The online version contains supplementary material available at 10.1007/s11136-024-03686-0.

## Plain English summary

Sleep disorders are common in the general population, and reduced quality of life (QoL) is a consequence of them, attributing to $25.5 billion in associated costs in 2019–20. No single instrument is available to measure all aspects of QoL or sleep outcomes relevant to people with sleep disorders in an economic evaluation (comparing the costs versus outcomes of interventions). Currently, a combination of a generic and sleep-specific questionnaires is commonly used to measure the benefits of sleep disorder interventions because using one or the other alone does not measure QoL with enough breadth. We compared two generic and three sleep-specific instruments to identify which instruments are appropriate in economic evaluations and if generic and sleep-specific questionnaires share an underlying relationship in their measurement of QoL or sleep outcomes. In the absence of a single ‘gold-standard’ questionnaire, our results support the combined use of a generic and sleep-specific instrument to measure QoL and sleep outcomes in sleep disorder patients. Therefore, depending on the research’s context, a combination of the generic EuroQoL 5-dimension 5-level and the sleep-specific Epworth Sleepiness Scale (for the broadest measurement of sleep outcomes) or Pittsburgh Sleep Quality Index (where bodily function is more important) is recommended.

## Introduction

Sleep disorders are prevalent in the general population despite being underdiagnosed and under-treated [1]. The incidence of the two most common sleep disorders in adults, obstructive sleep apnea (OSA) and insomnia, is estimated at approximately 1 in 7 [2] and 1 in 10 [3], respectively. The health consequences of sleep disorders include increased risk of cardiovascular disease, metabolic syndrome [4], reduced quality of life (QoL) [5], and mortality [6, 7]. Beyond the substantial burden of illness and loss of wellbeing, which put healthcare resources in high demand, sleep disorders pose a dramatic quantifiable economic burden [1] borne by patients, payers, and society [8]. Between 2019–20, the cost of sleep disorders in Australia was $35.4bil AUD, with 72% attributed to the non-financial reduction in wellbeing [1]. A cost incursion of this magnitude puts pressure on the distribution of limited public funds among competing healthcare interventions, making the knowledge of health economic aspects of intervention crucial for payers and decision-makers.

Economic evaluation is used by decision-makers to guide the cost-effective allocation of public funds and is recommended by decision-making bodies, including the Medical Benefits Advisory Committee (MSAC) in Australia, and the National Institute for Health and Clinical Excellence (NICE) in the United Kingdom [9, 10]. In addition to direct and indirect costs, outcomes such as QoL are a key health economic aspect of evaluating intervention benefits. Several generic preference and non-preference-based questionnaires have been used within sleep economic evaluations to measure QoL and other sleep outcomes. The EuroQoL 5-dimension 5-level (EQ-5D-5L) is amongst the most popular generic preference-based instruments used in such evaluations [13, 14]. An alternative instrument that, to our knowledge, has not been used in sleep economic evaluations is the ICEpop CAPability measure for Adults (ICECAP-A) [15], which focuses on capability-based QoL gains with a broader scope. It is however being used in other non-economic sleep studies [16, 17] Additionally, the ICECAP-A is considered a promising tool for future economic evaluations that extend beyond just health benefits [18]. Reimer and Flemons [19] have argued in support of using generic instruments to measure QoL in sleep- disordered populations, with consideration of physical, mental, and social function, symptom burden, and wellbeing. It is well-documented that sleep disturbance significantly impacts all aspects of QoL, often with a more pronounced effect on mental health [5], supporting the use of generic instruments broadly measuring the concept [19–21]. Measuring QoL with a broad scope is important, but capturing QoL attributes relevant to sleep disorder patients, such as sleep quality, fatigue, and energy, makes for a more effective measurement of intervention outcomes in sleep economic evaluations [11, 12]. Therefore, clinicians and researchers will often select condition-specific instruments with well-established validity because they focus on the unique aspects of a particular condition and can, therefore, detect subtle changes that might be missed by generic instruments [23]. While none of these instruments were developed to generate QALYs used in cost-utility analyses (CUA), and few measure QoL, they do take into account important factors relevant to populations dealing with sleep disorders and may have a place in sleep economic evaluations.

Our recent systematic review has shown that a combination of generic and sleep-specific instruments has been used in nearly half of the economic evaluations of sleep health interventions [14]. It is, however, unclear whether generic or sleep-specific instruments share an underlying, strong relationship in their measurement of QoL and other sleep outcomes. Therefore, this cross-sectional study explored the convergent and divergent validity between the generic EQ-5D-5L and ICECAP-A, and the sleep-specific 10-item Functional Outcomes of Sleep Questionnaire (FOSQ-10), Epworth Sleepiness Scale (ESS), and Pittsburgh Sleep Quality Index (PSQI) and their latent constructs in Australians who self-reporting having a sleep disorder. While the ESS and PSQI do not strictly measure QoL, they are commonly used screening tools, measuring daytime sleep propensity and sleep quality respectively. The conceptual overlap between instruments was also assessed using the International Classification of Functioning, Disability and Health (ICF) framework [24].

## Methods

### Sample

In this study, participants completed a structured questionnaire including a series of socio-demographic questions and five QoL instruments contemporaneously administered. Two instruments were generic: the EQ-5D-5L and ICECAP-A. Three instruments were sleep-specific: ESS, FOSQ-10, and the PSQI. The questionnaire was accessible via ‘PureProfile’, an online survey platform, on users’ dashboards from May 27th to August 9th 2022. Following advertisement of the study to PureProfile’s platform of users, informed consent was gained from all potential participants before proceeding to the questionnaire which gathered data from adults (aged 18 and above) who self-reported being diagnosed with a sleep disorder (participants were asked to specify which). Eligibility was based on predetermined quotas for various traits such as age, sleep disorder history, ethnicity, income, education, and employment status. Authentication criteria were applied to ensure legitimacy of respondents including CAPTCHA (Completely Automated Public Turing test to tell Computers and Humans Apart) test, open ends connectivity, internet protocol (IP) geolocation, and speeder limits. The goal was to secure a nationally representative sample of the Australian population. The target sample size was 1500, well exceeding the minimum recommended number of 50 participants for this kind of survey [25].

### Quality of life instruments

The EQ-5D-5L is a widely-used generic health-related QoL measure. Although not validated in sleep disordered populations, the EQ-5D-5L has shown excellent psychometric properties across a range of other disease populations and settings [26]. The EQ-5D, both 3- and 5- level, are the most frequently used generic instrument in economic evaluations within sleep disorders [14], though the EQ-5D-5L has reduced ceiling effects compared to its three-level counterpart [27]. It measures five dimensions: mobility, self-care, usual activities, pain/discomfort, and anxiety/depression each with five levels of impairment ranging from ‘no problem’ to ‘unable’ defining a possible 3125 health states. Using the Australian general population preference weights, a utility score ranging from −0.30 to 1 was attached to each health state [28].

The ICECAP-A is a generic measure of adult capability wellbeing measuring five dimensions: stability, attachment, achievement, autonomy, and enjoyment. Each attribute has four levels ranging from full to no capability to distinguish a possible 1024 capability states [15]. The algorithm based on a best–worst scaling method using UK population tariffs was applied to score the ICECAP-A and derive overall scores ranging from 0 (no capability) to 1 (full capability) [29].

The FOSQ-10 is a self-administered questionnaire assessing the impact of excessive somnolence on QoL in adults that is often applied in primary sleep disorders, particularly OSA. Shorter than its predecessor, the FOSQ-30 [30], the FOSQ-10 has performed well in recent studies [31, 32] and a newly established minimally important difference [33] improves its interpretability and applicability in practice. Due to the limited number of items per subscale (1 to 3), only the total score (ranging from 5 to 20) is recommended for use [34].

Applied across many sleep disorders and settings, the ESS is the most commonly used instrument for assessing excessive daytime sleepiness, which is a major consideration specifically in clinical decision-making concerning the diagnosis and management of OSA [35, 36]. It was also the most commonly applied sleep-specific instrument in economic evaluations [14]. Participants rate their propensity to doze or fall asleep on a four-point scale from 0 (never) to 3 (high chance) in eight different low-activity situations. Taking the sum of each item’s score, ranging from 0 to 24, an ESS score greater than 10 is indicative of excessive daytime sleepiness [37].

The PSQI is a self-report questionnaire used to discriminate between ‘poor’ and ‘good’ sleepers in clinical and non-clinical populations. Its application within sleep disorders is broad including sleep initiation and maintenance disorders and disorders of excessive somnolence [38]. Nineteen items generate 7 component scores (based on Likert response scored 0 to 3) and an overall ‘global’ score ranging from 0 to 21. Five additional items answered by a bed partner are excluded from scoring.

Higher scores for the EQ-5D-5L, ICECAP-A and FOSQ-10 indicated better outcomes, whereas the converse was true for the ESS and the PSQI.

### Conceptual overlap between instruments

The dimensions/items of all instruments were compared using the ICF to assess the conceptual overlap between the instruments [24]. The ICF is a multipurpose and comprehensive framework used to classify health and disability concepts within a biopsychosocial model of health, functioning, and disability [24]. Using the ICF framework, the instrument dimensions were categorised into two of four possible domains: (i) ‘body functions’ (measuring impairments of a) physiological and psychological functioning and b) anatomical functioning of body systems), and (ii) ‘activities and participation’ (measuring the full range of life areas including execution of tasks and management of life situations) [24]. Both domains were broken down into chapters which were further broken down into categories.

### Statistical analysis

Descriptive statistics (means, standard deviations, medians, interquartile ranges, frequencies) were generated. The distribution of the dimension/total scores of all QoL instruments were tested for normality using the Shapiro-Francia test [39]. All instrument dimension/total scores followed non-normal distribution (Shapiro-Francia test, p < 0.05), therefore, non-parametric tests of differences were applied (Wilcoxon-Mann Whitney, Kruskal Wallis, and Spearman’s correlation). Differences in dimension/total scores based on demographics (age band, gender, living arrangements, educational attainment) and self-rated QoL were tested using the Kruskal Wallis test.

Convergent validity between dimension/total scores of all instruments was assessed using Kendall’s Tau-B rank correlation significant at the 5% level, recommended for ordinal categorical data [40]. Correlations below 0.30 were considered weak, those between 0.30 and 0.50 moderate, and those above 0.50 strong. Strong absolute correlations between instruments, indicative of convergent validity, suggested they were measuring similar constructs [40]. We hypothesised that strong convergent validity would exist between items or dimensions belonging to the same ICF domains. Divergent validity was also assessed using Kendall’s Tau-B correlation. We hypothesised that divergent validity would be confirmed if items/dimensions belonging to different ICF domains had lower or no statistically significant correlation than those belonging to the same ICF chapter.

Exploratory Factor Analysis (EFA), incorporating Polychoric correlation matrices and iterative principal factor extraction to accommodate the ordinal and multivariate non-normal nature of the data (Mardia’s kurtosis p < 0.001 [41]), was conducted. Minimum average partials (MAP) [42] and Horn’s parallel analysis (PA) [43] were used to determine the optimal number of factors to retain. Promax oblique rotation with a kappa value of 3 was used to ensure realistic and statistically sound factor structures [44, 45]. The threshold for salient loadings was set at 0.32 as recommended in the literature [46]. The magnitude of loadings was interpreted as follows: ≥ 0.32 to < 0.45 was poor, ≥ 0.45 to < 0.55 was fair, ≥ 0.55 to < 0.63 was good, ≥ 0.63 to < 0.70 was very good, and ≥ 0.70 was excellent [47]. All candidate models were judged on their interpretability and theoretical sense to identify the most acceptable solution [48, 49]. Four sensitivity analyses testing the results’ robustness compared correlation matrices used (polychoric vs Pearson’s), factor extraction methods (iterative principal factor vs maximum likelihood), alternative rotation methods (promax vs oblimin), and outliers (using Mahalanobis distance (D^2^) [50].

There was no missing data to account for in our analyses. The assumed threshold for statistical significance in all analyses was 5% [51], and Stata version 15.1 (StataCorp, TX) was used to conduct all analyses.

### Ethical approval

This study adhered to the ethics guidelines set forth by the 1964 Helsinki Declaration and received approval from the Flinders University Social and Behavioural Ethics Committee.

### Consent

Informed consent was obtained from all individuals included in this study.

## Results

**Fig. 1:**
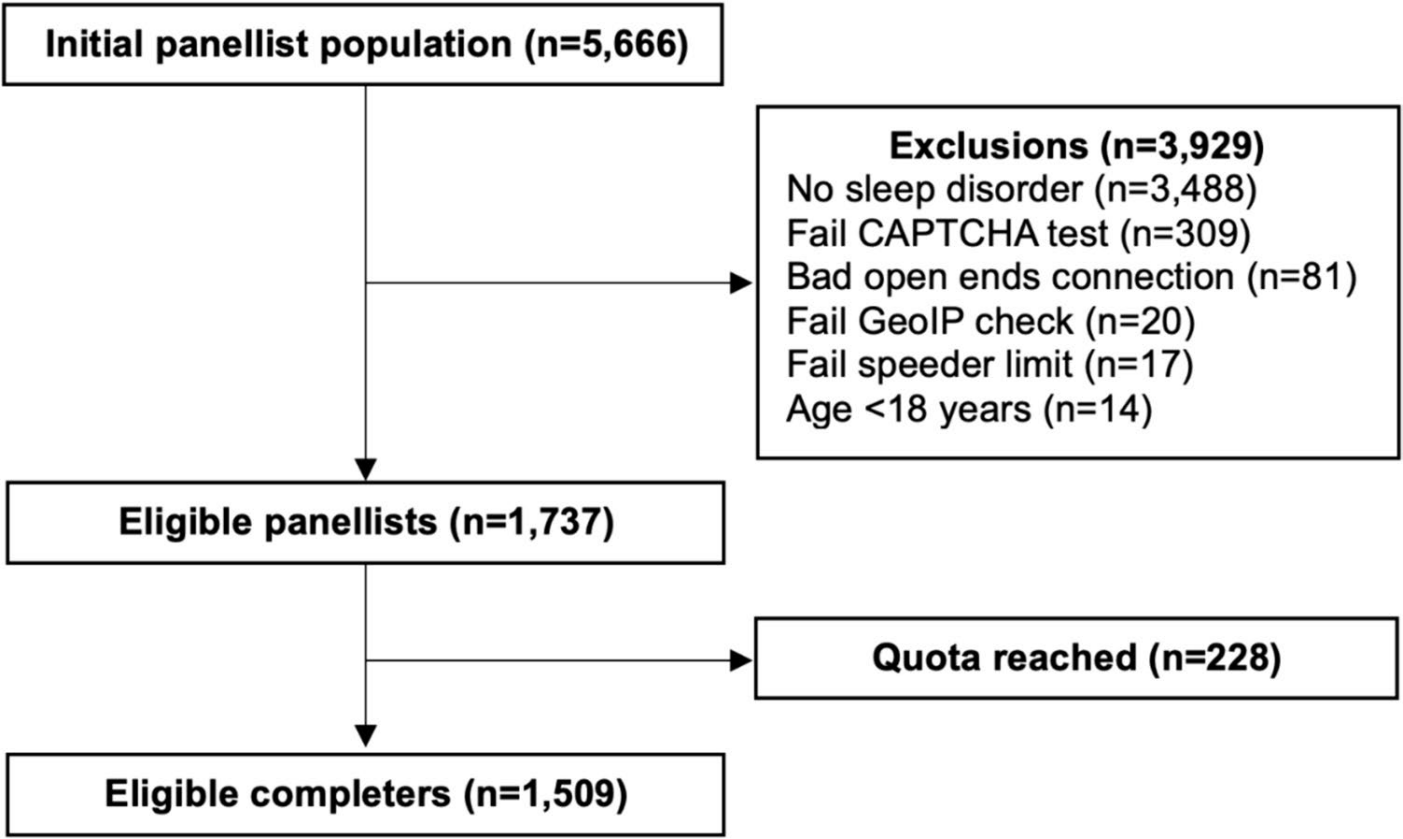
Sample selection flow chart. *CAPTCHA* Completely Automated Public Turing test to tell Computers and Humans Apart, *GeoIP* geographic internet protocol

Cohort selection is depicted in Fig. 1. Of the initial PureProfile panellist population (n = 5666), 1737 were eligible. The primary reason for exclusion was not having had a sleep disorder (n = 3488). After pre-determined quotas were reached, 1509 eligible panellists completed the survey. There were no partial completions or missing data.

### Sample characteristics

Participant characteristics are presented in Table 1. Participants’ ages ranged from 18 to 86 years (mean 46 and median 45). The sample consisted of nearly equal numbers of males and females. Self-rated QoL was indicated as average, good, or very good by 80.7% of participants. The frequency of self-reported sleep disorders is reported in Table 2. Bruxism was most commonly reported (n = 569, 37.7%), followed by insomnia (n = 553, 36.7%) and OSA (n = 467, 31.0%). Narcolepsy was least frequently reported (n = 41, 2.7%).

**Table 1:** Quality of life values for selected participant sociodemographics

Characteristic	N (%)	EQ-5D-5L median (IQR)	ICECAP-A median (IQR)	ESS median (IQR)	FOSQ-10 median (IQR)	PSQI median (IQR)
Whole sample (absolute score)	1,509 (100)	0.89 (0.78–0.96)	0.85 (0.69–0.94)	6 (3–9)	35 (28–38)	9 (6–12)
Whole sample (Z-score)^a^	1,509 (100)	0.00 (± 1.00)	0.00 (± 1.00)	0.00 (± 1.00)	0.00 (± 1.00)	0.00 (± 1.00)
Age						
18–24 years	188 (12.5)	0.89 (0.65–0.93)	0.81 (0.67–0.92)	8 (4–11)	29 (24–34)	10 (8–13)
25–34 years	292 (19.4)	0.92 (0.81–0.97)	0.86 (0.71–0.94)	7 (4–10	33 (25–37)	10 (7–13)
35–44 years	261 (17.3)	0.92 (0.85–0.97)	0.87 (0.70–0.97)	6 (3–10)	25 (28–38)	10 (7–13)
45–54 years	248 (16.4)	0.89 (0.76–0.96)	0.85 (0.66–0.92)	6 (3–9)	34 (27–38)	10 (7–14)
55–64 years	216 (14.3)	0.89 (0.78–0.96)	0.80 (0.61–0.92)	5 (2–8)	36 (31–39)	11 (7–14)
65–74 years	233 (15.4)	0.89 (0.80–0.96)	0.87 (0.81–0.92)	4 (2–8)	38 (34–40)	9 (6–12)
75 + years	69 (4.6)	0.92 (0.80–0.93)	0.87 (0.81–0.92)	5 (3–8)	38 (35–40)	9 (6–12)
Prefer not to say	2 (0.1)	0.86 (0.76–0.96)	0.90 (0.79–1.00)	12 (7–16)	22 (14–30)	10 (5–14)
* P*-value^b^		** < 0.01**	** < 0.01**	** < 0.01**	** < 0.01**	0.19
Gender						
Male	753 (49.9)	0.92 (0.81–0.97)	0.88 (0.70–0.95)	6 (3–10)	35 (29–39)	10 (7–12)
Female	753 (49.9)	0.89 (0.76–0.93)	0.84 (0.67–0.92)	5 (3–9)	34 (27–38)	10 (8–14)
Other	1 (0.1)	0.62 (0.63–0.63)	0.92 (0.92–0.92)	13 (13–13)	33 (33–33)	missing
Prefer not to say	2 (0.1)	0.98 (0.96—1.00)	0.65 (0.51–0.79)	7 (7–7)	34 (30–37)	7 (5–8)
*P*-value^b^		** < 0.01**	** < 0.01**	**0.02**	** < 0.01**	** < 0.01**
Aboriginal or torres strait islander status						
No	1423 (94.3)	0.92 (0.80–0.96)	0.85 (0.69–0.94)	6 (3–9)	35 (29–38)	10 (7–13)
Yes, aboriginal	73 (4.8)	0.81 (0.49–0.92)	0.79 (0.57–0.93)	8 (4–13)	28 (22–34)	11 (8–14)
Yes, torres strait islander	2 (0.1)	0.36 (0.08–0.65)	0.52 (0.51–0.53)	9 (7–10)	28 (24–31)	10 (8–11)
Yes, both aboriginal and torres strait islander	5 (0.3)	0.34 (0.20–0.76)	0.76 (0.07–0.87)	16 (14–19)	14 (14–29)	12 (9–14)
Prefer not to say	6 (0.4)	0.81 (0.60–0.96)	0.81 (0.67–0.88)	8 (4–15)	32 (20–36)	8 (7–15)
*P*-value^b^		** < 0.01**	0.05	** < 0.01**	** < 0.01**	0.25
Country of birth						
Australia	1215 (80.5)	0.89 (0.76–0.96)	0.85 (0.67–0.84)	6 (3–10)	34 (27–38)	10 (7–13)
Not australia	283 (18.8)	0.92 (0.85–0.96)	0.87 (0.70–0.94)	6 (3–9)	36 (30–39)	9 (7–12)
Prefer not to say	11 (0.7)	0.92 (0.86–0.97)	0.80 (0.62–0.97)	5 (0–9)	39 (32–40)	11 (10–17)
*P*-value^b^		** < 0.01**	0.24	0.29	** < 0.01**	**0.03**
Living arrangements						
On your own	379 (25.1)	0.89 (0.76–0.96)	0.81 (0.64–0.92)	6 (3–10)	35 (29–38)	10 (7–13)
With your spouse	794 (52.6)	0.92 (0.82–0.96)	0.88 (0.76–0.95)	6 (3–9)	35 (29–39)	10 (7–12)
With other family	283 (18.8)	0.89 (0.71–0.93)	0.79 (0.60–0.90)	7 (3–10)	33 (26–37)	10 (8–13)
With others‚ not family	51 (3.4)	0.92 (0.61–0.93)	0.81 (0.63–0.94)	6 (3–11)	35 (28–38)	10 (7–13)
Prefer not to say	2 (0.1)	0.71 (0.42–1.00)	0.39 (0.00–0.79)	3 (1–4)	30 (20–40)	21 (21–21)
*P*-value^b^		** < 0.01**	** < 0.01**	0.14	** < 0.01**	**0.01**
Education level						
Primary or some secondary school (e.g. 8th grade or less)	20 (1.3)	0.78 (0.35–0.89)	0.75 (0.51–0.85)	9 (6–13)	28 (23–31)	10 (9–15)
Some high school, but did not graduate	132 (8.8)	0.88 (0.67–0.93)	0.85 (0.61–0.91)	6 (3–9)	36 (30–39)	10 (7–14)
High school graduate or HSC	262 (17.4)	0.92 (0.76–0.96)	0.85 (0.69–0.94)	5 (3–9)	35 (29–38)	10 (7–13)
Tafe graduate (e.g., Certificate, diploma)	460 (30.5)	0.89 (0.72–0.93)	0.85 (0.64–0.92)	6 (3–9)	35 (28–38)	10 (7–13)
University graduate (e.g., Bachelor’s degree)	475 (31.5)	0.92 (0.85–0.97)	0.85 (0.70–0.95)	6 (3–10)	34 (27–38)	9 (6–12)
More than 4-year university degree (e.g. Masters, PhD)	157 (10.4)	0.92 (0.88–1.00)	0.89 (0.76–0.97)	6 (3–10)	36 (30–39)	9 (7–12)
Prefer not to say	3 (0.2)	1 (0.76–1.00)	1 (1.00–1.00)	13 (0–16)	40 (14–40)	14 (8–22)
*P*-value^b^		** < 0.01**	** < 0.01**	**0.03**	**0.01**	**0.01**
Self-rated quality of life						
Very good	80 (5.3)	0.97 (0.92–1.00)	0.97 (0.88–1.00)	5 (2–10)	38 (31–40)	6 (4–10)
Good	532 (35.3)	0.93 (0.89–0.97)	0.90 (0.83–0.97)	5 (3–9)	36 (31–39)	8 (6–11)
Average	606 (40.2)	0.89 (0.81–0.93)	0.85 (0.69–0.92)	6 (3–10)	34 (27–38)	10 (8–13)
Poor	225 (14.9)	0.77 (0.57–0.89)	0.67 (0.51–0.81)	6 (3–11)	32 (26–36)	12 (10–15)
Very poor	66 (4.4)	0.58 (0.28–0.77)	0.43 (0.30–0.71)	9 (5–12)	29 (20–34)	14 (11–16)
*P*-value^b^		** < 0.01**	** < 0.01**	** < 0.01**	** < 0.01**	** < 0.01**

*EQ-5D-5L* EuroQoL 5-Dimension 5-Level, *ICECAP-A* ICEpop CAPability measure for Adults, *ESS* Epworth Sleepiness Scale, *FOSQ-10* 10-item Functional Outcomes of Sleep Questionnaire, *PSQI* Pittsburgh Sleep Quality Index

^a^Utility and summary scores were power transformed to follow a normal distribution (using the square transformation) before converting them to Z-scores. Results are reported for mean (± standard deviation)

^b^Kruskal-Wallis test, 5% significance threshold

**Table 2:** Frequency of self-reported sleep disorders

Sleep disorder*	N (%)
OSA	467 (31.0)
Insomnia	553 (36.7)
Narcolepsy	41 (2.7)
Restless legs syndrome	292 (19.4)
Bruxism	569 (37.7)
REM sleep behaviour disorder	53 (3.5)
Parasomnia	169 (11.2)
Delayed sleep phase disorder	67 (4.4)

^*^Not mutually exclusive

*OSA* obstructive sleep apnea, *REM* rapid eye movement

### Quality of life instrument scores

The distribution of total (ESS, FOSQ, PSQI) and utility (EQ-5D-5L, ICECAP-A) scores across participant characteristics is shown in Table 1. Median (IQR) EQ-5D-5L and ICECAP-A utility scores were 0.89 (0.78–0.96) and 0.85 (0.69–0.94) respectively. Median (IQR) total scores on the ESS, FOSQ-10, and PSQI were 6 (3–9), 35 (28–38), and 9 (6–12), respectively. For all five instruments, there was a statistically significant relationship between utility/total score and self-rated QoL, suggesting that all instruments were able to discriminate according to self-rated QoL (Kruskal Wallis test, p < 0.01). The distribution of utility/total scores for all instruments across all dimension levels of the EQ-5D-5L and ICECAP-A are presented in the supplementary material Tables S1 and S2.

### Conceptual overlap between instruments

Table 3 presents the conceptual overlap according to the ICF framework between all instrument items/dimensions. When the generic instruments were compared to sleep-specific instruments, the most overlap between the two types of instruments was seen in their capture of the ICF ‘activities and participation’ domain. Three of the five (60%) EQ-5D-5L dimensions (‘usual activities’, ‘mobility’ and ‘self-care’) and all the ICECAP dimensions captured this domain. In comparison, all the ESS items, eight of the 10 (80%) FOSQ-10 items (i.e., all except ‘concentration’ and ‘remembering’), and two of the seven (29%) of the PSQI items (‘daytime dysfunction’ and ‘use of sleep medication’) also captured this domain. There was, however, less overlap in the way the two types of instruments captured the ICF ‘body functions’ domain. Only two of five (40%) EQ-5D-5L dimensions (‘anxiety/depression’ and ‘pain/discomfort’) and none of the ICECAP dimensions captured this domain. In contrast, all of the ESS and FOSQ-10, and six of the seven (86%) of the PSQI items (i.e., all except ‘use of sleep medication’) captured this domain.

**Table 3:** Classification of the EQ-5D-5L, ICECAP-A, ESS, FOSQ-10, and PSQI items/dimensions according to the International Classification of Functioning and Disability Functioning (ICF) framework

International Classification of Functioning, Disability and Health (ICF) classifications		Quality of life instruments				
ICF domain	ICF chapter	EQ-5D-5L 5 dimensions	ICECAP-A 5 dimensions	ESS 8 items	FOSQ-10 10 items	PSQI 7 dimensions
Body function	Chapter b1: Mental functions	Anxiety/ depression	N/A	Sitting and reading	Concentration	Sleep quality
				Watching TV	Remembering	Sleep latency
				Sitting, inactive	Finishing a meal	Sleep duration
				Car passenger	Working on a hobby	Sleep efficiency
				Lying down to rest	Housework	Sleep disturbances
				Sitting and talking	Operating motor vehicle (short)	Daytime dysfunction
				Sitting quietly	Operating motor vehicle (long)	
				In a car	Difficulty completing tasks	
					Managing financial affairs	
					Performing work	
	Chapter b2: Sensory functions and pain	Pain/ discomfort	N/A	N/A	N/A	Sleep disturbances
Activities and participation	Chapter d1: Learning and applying knowledge	N/A	Achievement	Watching TV	N/A	N/A
	Chapter d2: General tasks and demands	Usual activities	Security	In a car	Working on a hobby	Daytime dysfunction
					Housework	
					Operating motor vehicle (short)	
					Operating motor vehicle (long)	
			Autonomy		Difficulty completing tasks	
			Achievement		Performing work	
	Chapter d3: Communication	N/A	N/A	Sitting and talking	N/A	N/A
	Chapter d4: Mobility	Mobility	N/A	Sitting and reading	Operating motor vehicle (short)	Daytime dysfunction
				Car passenger	Operating motor vehicle (long)	
				In a car	Difficulty completing tasks	
	Chapter d5: Self-care	Self-care	Autonomy	Lying down to rest	Finishing a meal	Use of sleep medication
	Chapter d6: Domestic life	Usual activities	Security	N/A	Housework	N/A
			Autonomy			
	Chapter d7: Interpersonal interactions and relationships	Usual activities	Autonomy	Sitting and talking	N/A	Daytime dysfunction
			Attachment			
	Chapter d8: Major life areas	Usual activities	Achievement	N/A	Managing financial affairs	N/A
					Performing work	
	Chapter d9: Community, social and civic life	Usual activities	Enjoyment	Sitting and reading	Working on a hobby	Daytime dysfunction
				Sitting and talking		
				Watching TV		
				Sitting, inactive		
				Sitting quietly		

*EQ-5D-5L* EuroQoL 5-Dimension 5-Level, *ICECAP-A* ICEpop CAPability measure for Adults, *ESS* Epworth Sleepiness Scale, *FOSQ-10* 10-item Functional Outcomes of Sleep Questionnaire, *PSQI* Pittsburgh Sleep Quality Index

### Convergent and divergent validity

Results of the assessment of validity between the dimensions and item scores of all instruments is presented in supplementary Table S4. Although correlations existed in the direction expected between items/dimensions of different instruments hypothesised to measure similar constructs, and were statistically significant in most cases, they were weak to moderate at best. Between the EQ-5D-5L or ICECAP-A and all sleep-specific instruments, statistically significant absolute correlation ranged from 0.06 (‘car passenger’ [ESS] vs ‘pain’ [EQ-5D-5L]) to 0.35 (‘daytime dysfunction’ [PSQI] vs ‘anxiety/depression’ [EQ-5D-5L]). The EQ-5D-5L and FOSQ-10 shared the most moderate correlations. Indicative of poor divergent validity, correlation coefficients between items/dimensions of generic versus sleep-specific instruments hypothesised to belong to different ICF domains ranged from 0.05 (‘sleep duration’ [PSQI] vs ‘usual activities’ [EQ-5D-5L]) to 0.33 (‘sleep quality’ [PSQI] and ‘enjoyment’ [ICECAP-A]) (Kendall’s Tau-B, p < 0.05).

### Exploratory factor analysis

EFA results are shown in Table 4, depicting loadings of instrument items/dimensions on to the latent factors. Parallel analysis suggested 5 factors be retained, whereas MAP suggested 6 factors be retained (supplementary Tables S5 and S6). Root mean squared residual (RMSR) indicated a close fit of the model (RMSR = 0.041). Assessing all candidate models’ fit showed that, compared to a 6-factor solution, a 5-factor solution had fewer cross-loadings, and removing Factor 6 did not change the RMSR suggesting that a 5-factor solution was simpler. Before rotation, Factors 1 and 2 explained 37.1% and 11.4% of total variance respectively while Factors 3, 4, and 5 collectively explained 14.5%. Nearly two thirds (64.9%) of salient loadings were excellent in magnitude. All items of the FOSQ-10 had excellent magnitude salient loadings onto Factor 1. In contrast, the PSQI ‘daytime dysfunction’ had fair magnitude, while ESS items ‘sitting and talking’ and ‘in a car’ showed poor magnitude. Only items of the ESS loaded on to Factor 2, with 5 of 8 items of excellent magnitude. Factor 3 was loaded onto by all ICECAP-A dimensions, 4 of 5 with an excellent magnitude, and one EQ-5D-5L item (‘anxiety/depression’) with fair magnitude respectively. The ICECAP-A dimension ‘autonomy’ had cross-loading on to Factors 3 and 4 of poor and fair magnitude. All EQ-5D-5L dimensions except ‘anxiety/depression’ loaded saliently on to Factor 5 with good or excellent magnitude. All PSQI components except ‘use of sleep medication’ and ‘daytime dysfunction’ loaded saliently on to Factor 5. The components ‘sleep quality’, ‘sleep duration’, and ‘sleep efficiency’ components loaded with a very good to excellent magnitude. PSQI ‘use of sleep medications’ was the only dimension/item not meeting the salient loading threshold.

**Table 4:** Results of exploratory factor analysis showing factor loadings of the EQ-5D-5L, ICECAP-A and PSQI dimensions, and ESS and FOSQ-10 items^a^

Variable	Factor 1	Factor 2	Factor 3	Factor 4	Factor 5	Uniqueness
EQ-5D-5L dimensions						
Mobility				0.86		0.26
Self-care				0.77		0.19
Usual activities				0.74		0.21
Pain/discomfort				0.55		0.47
Anxiety/depression			0.51			0.54
ICECAP-A dimensions						
Stability			0.86			0.26
Attachment			0.74			0.48
Autonomy			0.39	0.46		0.46
Achievement			0.76			0.30
Joy			0.79			0.30
ESS items						
Sitting and reading		0.77				0.41
Watching TV		0.76				0.47
Sitting, inactive		0.77				0.28
Car passenger		0.73				0.40
Lying down to rest		0.62				0.61
Sitting and talking	−0.36	0.55				0.26
Sitting quietly		0.72				0.33
In a car	−0.39	0.54				0.29
FOSQ-10 items						
Concentration	0.73					0.32
Remembering	0.74					0.34
Finishing a meal	0.84					0.27
Working on a hobby	0.78					0.29
Housework	0.75					0.32
Operating motor vehicle (short distance)	0.91					0.19
Operating a motor vehicle (long distance)	0.77					0.37
Difficulty completing tasks	0.87					0.22
Managing financial affairs	0.90					0.25
Performing work	0.84					0.23
PSQI components						
Sleep quality					0.66	0.38
Sleep latency					0.53	0.60
Sleep duration					0.82	0.45
Sleep efficiency					0.81	0.46
Sleep disturbances					0.38	0.59
Use of sleep medication						0.78
Daytime dysfunction	−0.51					0.42
Total variance explained by factor (%)	37.1	11.4	5.6	5.1	3.8	n/a
Determinant	*p* < 0.001					
Bartlett’s test of sphericity	*X*^2^ = 28151.71, *p* < 0.001					
Kaiser–Meyer–Olkin measure of sampling adequacy	0.94					

*EQ-5D-5L* EuroQoL 5-Dimension 5-Level, *ICECAP-A* ICEpop CAPability measure for Adults, *ESS* Epworth Sleepiness Scale, *FOSQ* 10-item Functional Outcomes of Sleep Questionnaire, *PSQI* Pittsburgh Sleep Quality Index, *n/a* not applicable

^a^Blanks represent absolute loading < 0.32, the assumed threshold for salient loading

### Sensitivity analysis

Comparing polychoric and Pearson’s correlation matrices used for EFA, the determinant, Bartlett’s test of sphericity, and KMO results (Tables 4 and S7) show that either correlation matrix was similarly appropriate. Factor extraction using maximum likelihood was similarly parsimonious to iterative principal factor extraction, Factors 1 and 2 explained 48.2% and 48.5% of total variance before rotation respectively with similar uniqueness values. Promax and oblimin rotated solutions shared the same factor loadings and loading magnitudes. Based on the critical value (D^2^ = 59.7) with 30 degrees freedom and using the conservative p < 0.001 significance level, 146 observations were identified as potential outliers. EFA excluding outliers shared the same close fit (RMSR = 0.041) with identical factor loadings of similar magnitude (supplementary Table S8) and was also robust to extraction and rotation methods. Therefore, our EFA results were robust across correlation matrices, extraction method, rotation, and outliers.

## Discussion

This study is the first to report an empirical comparison of two generic and three sleep-specific metrics using data from a sample of the Australian general population previously diagnosed with a sleep disorder(s). Weak to moderate convergence between the generic versus sleep-specific instruments supports their application in parallel versus as alternatives within an economic evaluation. Our results suggest that, due to their wider coverage of QoL concepts as summarised by the ICF framework, the EQ-5D-5L in conjunction with the ESS or PSQI are the most appropriate instruments amongst those used in this study to apply dependent on context, content coverage, and latent constructs in economic evaluations and other similar sleep evaluations.

Based on ICF chapters that instrument items/dimensions were hypothesised to load on, statistically significant correlations of items/dimensions measuring the same constructs was moderate at best. The weak to moderate convergence observed between items/dimensions of generic and sleep-specific instruments supports the practice of using both types of instrument in parallel [14] to adequately measure QoL in sleep disorders in general within economic evaluation. An explanation for the weak to moderate magnitudes of correlation observed is that the ICF framework may not adequately capture unaccounted-for, unrecognised, and/or unmeasured latent constructs shared between items/dimensions [52]. For example, ‘sleep quality’ (PSQI) is not only related to ‘anxiety/depression’ (EQ-5D-5L), but several confounding external factors including the physical and social environment [53, 54] and comorbid chronic conditions [55]. Consequently, the strength of association between two items/dimensions hypothesised to measure the same construct is reduced due to the confounding of variables unaccounted for. Similar findings have been reported elsewhere [56, 57].

The robust 5-factor solution EFA showed that each instrument loaded primarily onto its own factor with the exceptions of ‘anxiety/depression’ (EQ-5D-5L) and ‘daytime dysfunction’ (PSQI). Factor 1 was loaded onto by all FOSQ-10 items, 2 ESS items, and 1 PSQI component. Given these items primarily measure daytime functional limitations precipitating from somnolence or poor sleep, Factor 1 can be characterised as ‘daytime dysfunction’. Loading onto Factor 2 was exclusive to ESS items, which Johns [37] posited represents ‘somnoficity’. However, cross-loadings of items 6 (‘sitting and talking’) and 8 (‘in a car’) suggests a single-factor solution is inadequate to describe the latent constructs of the ESS, also shown elsewhere [58]. In fact, Smith et al. [58] found items 6 and 8 of the ESS to be poor measures of the single ‘somnoficity’ construct with few participants reporting ‘a high chance of dozing’ in these situations. Therefore, an alternative characterisation of Factor 2 is required. Evidence suggests that the subjective daytime symptoms of OSA, as measured by the ESS [37], are better conceptualised as ‘fatigue’ rather than ‘somnoficity’ or sleep propensity [58, 59]; hence ‘fatigue’ is a better descriptor of Factor 2. Factor 3 can be characterised as measuring ‘wellbeing’, a broader concept capturing attributes of QoL that reflects the item loadings [60] and is in line with the development approach of the ICECAP-A [15]. Cross-loading of ‘autonomy’ (ICECAP-A) on to Factors 3 and 4 supports the position that not all aspects of Factor 3 are ‘psychosocial’ [60], as posited by Davis et al. [61]. Based on the dominance of items assessing physical functioning loading onto Factor 4, it can be characterised as measuring ‘physical functioning’. These findings and characterisations are commensurate with previous EFAs comparing the EQ-5D-3L with the ICECAP-A [60] and ICECAP-O [61]. Further, the factor loadings of the EQ-5D-5L in this study were the same as those reported for the EQ-5D-3L [60, 61], showing that the additional dimension levels do not change the instrument’s measurement of latent constructs. All PSQI items requiring a subjective assessment of sleep loaded on to Factor 5, as such it is plausible to characterise this factor as ‘perceived sleep quality’. Failure of ‘use of sleep medication’ to load saliently onto any factor is consistent with another EFA [62].

In the absence of a ‘gold standard’ instrument capturing relevant attributes of QoL in sleep disorders, which combination of instruments to use within economic evaluations in general sleep disorder populations should be determined based on study aim(s), content coverage, and latent constructs. The economic evaluation results should enable standardised comparisons across other non-sleep disorders, as in a CUA or CEA. In the case where utility scores are required, as in a CUA, the EQ-5D-5L is one of the instruments recommended by decision-making bodies such as NICE and MSAC [9, 10]. While the EQ-5D-5L remains the most applied instrument in economic evaluations of sleep interventions, the ICECAP-A would be more appropriate in contexts where there is a need to reflect changes in wellbeing-related capability. However, some measurement properties of generic QoL instruments in sleep disorder populations have yet to be demonstrated. Therefore, using a complementary sleep-specific instrument in parallel is required, and ICF framework coverage and latent constructs are additional considerations informing instrument choice. The ICF ‘activities and participation’ domain was captured more than the ‘body functions’ domain. Between the generic EQ-5D-5L and ICECAP-A, the former covers more of the ICF framework (measuring two ICF domains) and two latent constructs (Factors 3 [‘wellbeing’] and 4 [‘physical health’]), suggestive of broader coverage of QoL aspects. Of the sleep-specific instruments, the ICF framework was most broadly covered by the ESS, but the PSQI better measured the ‘body functions’ domain. The consideration of latent constructs shows that ‘daytime dysfunction’ (Factor 1) is common to all sleep-specific instruments, however the ESS and PSQI measure one additional factor each, ‘fatigue’ (Factor 2) and ‘perceived sleep quality’ (Factor 5) respectively. Within sleep disorder cohorts in general, the combination of generic and sleep-specific instrument should be guided by the context in which they will be applied. From the selection of instruments used in this study, a full economic evaluation should employ the EQ-5D-5L as the generic instrument to allow QALY calculation. The accompanying sleep-specific instrument depends on aspects of QoL that need to be measured, with the ESS offering the broadest content coverage and measurement of latent constructs. Although, the PSQI would be more appropriate in contexts where measuring ‘body functions’ is of import.

Several limitations of this study merit note. Data used in this study was collected using a cross-sectional study design where future research should consider a longitudinal study design. Such study design would allow the evaluation of which instruments are most responsive and best capture clinically important changes over time, supporting the assessment of incremental effectiveness in economic evaluation [63, 64]. Further, expanding the comparisons to include other generic and/or sleep-specific instruments should be considered because they may be more applicable in the assessment of sleep health interventions. For example the newly-developed SF-6Dv2 [65] has shown comparative validity to the EQ-5D-5L [66] but is more sensitive to clinical improvements in OSA patients [67], and a recently derived value set enables the calculation of QALYs for health technology assessment [68]. Other jurisdictions should extrapolate our results cautiously given they are based on a nationally representative sample of Australians who self-reported having a sleep disorder. As such, replication of this study in other countries is appropriate.

## Conclusion

In the absence of a gold standard, neither a generic nor sleep-specific instrument alone is sufficient for measuring outcomes in general populations diagnosed with a sleep disorder(s) within an economic or other evaluations. Therefore, a combination of both is required. The context of the research to be done should guide the instrument choice.

### Supplementary Information

Below is the link to the electronic supplementary material.

Supplementary file1 (DOCX 97 KB)

Supplementary file2 (DOCX 52 KB)

## Data Availability

The dataset analysed in support of the conclusions of this study is not publicly available but, upon reasonable request to the corresponding author, may be made available in accordance with the ethical approval provided by Flinders University Social and Behavioural Ethics Committee.
