# Supplementary material for: An empirical comparison of sleep-specific versus generic quality of life instruments among Australians with sleep disorders
Source: Qual Life Res. 2024 Jun 24;33(8):2261–74. doi: 10.1007/s11136-024-03686-0 (PMC11286652; doi:10.1007/s11136-024-03686-0)
Supplement: Supplementary file 1 — Supplementary file1 (DOCX 97 KB) [file 11136_2024_3686_MOESM1_ESM.docx]

STROBE Statement—checklist of items that should be included in reports of observational studies

|  | Item No. | Recommendation | Page  No. | Relevant text from manuscript |
| --- | --- | --- | --- | --- |
| **Title and abstract** | 1 | (a) Indicate the study’s design with a commonly used term in the title or the abstract | 1 (Abstract) | “In Australian adults diagnosed with a sleep disorder(s), this cross-sectional study compares the empirical relationships between two generic QoL instruments… and three sleep-specific metrics…” |
|  |  | (b) Provide in the abstract an informative and balanced summary of what was done and what was found | 1-2 | “Convergent and divergent validity between item/dimension scores was examined using Kendall’s Tau-B correlation, with correlations below 0.30 considered weak, between 0.30 and 0.50 moderate and those above 0.50 strong (indicating that instruments were measuring similar constructs). Exploratory factor analysis (EFA) was conducted to identify shared underlying constructs. … Convergent validity between dimensions/items of different instruments was weak to moderate. A 5-factor EFA solution, representing ‘daytime dysfunction’, ‘fatigue’, ‘wellbeing’, ‘physical health’, and ‘perceived sleep quality’, was simplest with close fit and fewest cross-loadings. Each instrument’s dimensions/items primarily loaded onto their own factor, except for the EQ-5D-5L and PSQI. Nearly two-thirds of salient loadings were of excellent magnitude (0.72 to 0.91).” |
| Introduction | | | |  |
| Background/rationale | 2 | Explain the scientific background and rationale for the investigation being reported | 4-5 | “Several generic preference and non-preference-based questionnaires have been used within sleep economic evaluations to measure QoL and other sleep outcomes. The EuroQoL 5-dimension 5-level (EQ-5D-5L) is amongst the most popular generic preference-based instruments used in such evaluations [13, 14]. An alternative instrument that, to our knowledge, has not been used in sleep economic evaluations is the ICEpop CAPability measure for Adults (ICECAP-A) [15], which focuses on capability-based QoL gains with a broader scope. It is however being used in other non-economic sleep studies[16, 17] Additionally, the ICECAP-A is considered a promising tool for future economic evaluations that extend beyond just health benefits [18]. Reimer and Flemons [19] have argued in support of using generic instruments to measure QoL in sleep- disordered populations, with consideration of physical, mental, and social function, symptom burden, and wellbeing. It is well-documented that sleep disturbance significantly impacts all aspects of QoL, often with a more pronounced effect on mental health[5], supporting the use of generic instruments broadly measuring the concept [19, 20, 21]. Measuring QoL with a broad scope is important, but capturing QoL attributes relevant to sleep disorder patients, such as sleep quality, fatigue, and energy, makes for a more effective measurement of intervention outcomes in sleep economic evaluations [11, 12]. Therefore, clinicians and researchers will often select condition-specific instruments with well-established validity because they focus on the unique aspects of a particular condition and can, therefore, detect subtle changes that might be missed by generic instruments [23]. While none of these instruments were developed to generate QALYs used in cost-utility analyses (CUA), and few measure QoL, they do take into account important factors relevant to populations dealing with sleep disorders and may have a place in sleep economic evaluations.” |
| Objectives | 3 | State specific objectives, including any prespecified hypotheses | 5 | “Our recent systematic review has shown that a combination of generic and sleep-specific instruments has been used in nearly half of the economic evaluations of sleep health interventions [14]. It is, however, unclear whether generic or sleep-specific instruments share an underlying, strong relationship in their measurement of QoL and other sleep outcomes. Therefore, this cross-sectional study explored the convergent and divergent validity between the generic EQ-5D-5L and ICECAP-A, and the sleep-specific FOSQ-10, ESS, and PSQI and their latent constructs in Australians who self-reporting having a sleep disorder. While the ESS and PSQI do not strictly measure QoL, they are commonly used screening tools, measuring daytime sleep propensity and sleep quality respectively. The conceptual overlap between instruments was also assessed using the International Classification of Functioning, Disability and Health (ICF) framework [24].” |
| Methods | | | |  |
| Study design | 4 | Present key elements of study design early in the paper | 5 | “In this study, participants completed a structured questionnaire including a series of socio-demographic questions and five QoL instruments contemporaneously administered. Two instruments were generic: the EQ-5D-5L and ICECAP-A. Three instruments were sleep-specific: ESS, FOSQ-10, and the PSQI.” |
| Setting | 5 | Describe the setting, locations, and relevant dates, including periods of recruitment, exposure, follow-up, and data collection | 5 | “The questionnaire was accessible via 'PureProfile', an online survey platform, on users’ dashboards from May 27th to August 9th 2022. Following advertisement of the study to PureProfile’s platform of users, informed consent was gained from all potential participants before proceeding to the questionnaire which gathered data from adults (aged 18 and above) who self-reported being diagnosed with a sleep disorder (participants were asked to specify which).” |
| Participants | 6 | (a) ~~Cohort study—Give the eligibility criteria, and the sources and methods of selection of participants. Describe methods of follow-up~~  ~~Case-control study—Give the eligibility criteria, and the sources and methods of case ascertainment and control selection. Give the rationale for the choice of cases and controls~~  Cross-sectional study—Give the eligibility criteria, and the sources and methods of selection of participants | 5-6 | “Eligibility was based on predetermined quotas for various traits such as age, sleep disorder history, ethnicity, income, education, and employment status. Authentication criteria were applied to ensure legitimacy of respondents including CAPTCHA (Completely Automated Public Turing test to tell Computers and Humans Apart) test, open ends connectivity, internet protocol (IP) geolocation, and speeder limits. The goal was to secure a nationally representative sample of the Australian population. The target sample size was 1,500, well exceeding the minimum recommended number of 50 participants for this kind of survey [25].” |
|  |  | (b) Cohort study—For matched studies, give matching criteria and number of exposed and unexposed  Case-control study—For matched studies, give matching criteria and the number of controls per case | N/A | This study is cross-sectional |
| Variables | 7 | Clearly define all outcomes, exposures, predictors, potential confounders, and effect modifiers. Give diagnostic criteria, if applicable | 5 | “In this study, participants completed a structured questionnaire including a series of socio-demographic questions and five QoL instruments contemporaneously administered. Two instruments were generic: the EQ-5D-5L and ICECAP-A. Three instruments were sleep-specific: ESS, FOSQ-10, and the PSQI.” |
| Data sources/ measurement | 8* | For each variable of interest, give sources of data and details of methods of assessment (measurement). Describe comparability of assessment methods if there is more than one group | 5-7 | “In this study, participants completed a structured questionnaire including a series of socio-demographic questions and five QoL instruments contemporaneously administered. Two instruments were generic: the EQ-5D-5L and ICECAP-A. Three instruments were sleep-specific: ESS, FOSQ-10, and the PSQI.…. The EQ-5D-5L is a widely-used generic health-related QoL measure. Although not validated in sleep disordered populations, the EQ-5D-5L has shown excellent psychometric properties across a range of other disease populations and settings [26]. The EQ-5D, both 3- and 5- level, are the most frequently used generic instrument in economic evaluations within sleep disorders [14], though the EQ-5D-5L has reduced ceiling effects compared to its three-level counterpart [27]. It measures five dimensions: mobility, self-care, usual activities, pain/discomfort, and anxiety/depression each with five levels of impairment ranging from ‘no problem’ to ‘unable’ defining a possible 3125 health states. Using the Australian general population preference weights, a utility score ranging from -0.30 to 1 was attached to each health state [28].  The ICECAP-A is a generic measure of adult capability wellbeing measuring five dimensions: stability, attachment, achievement, autonomy, and enjoyment. Each attribute has four levels ranging from full to no capability to distinguish a possible 1024 capability states [15]. The algorithm based on a best-worst scaling method using UK population tariffs was applied to score the ICECAP-A and derive overall scores ranging from 0 (no capability) to 1 (full capability) [29].  The FOSQ-10 is a self-administered questionnaire assessing the impact of excessive somnolence on QoL in adults that is often applied in primary sleep disorders, particularly OSA. Shorter than its predecessor, the FOSQ-30 [30], the FOSQ-10 has performed well in recent studies [31, 32] and a newly established minimally important difference [33] improves its interpretability and applicability in practice. Due to the limited number of items per subscale (1 to 3), only the total score (ranging from 5 to 20) is recommended for use [34].  Applied across many sleep disorders and settings, the ESS is the most commonly used instrument for assessing excessive daytime sleepiness, which is a major consideration specifically in clinical decision-making concerning the diagnosis and management of OSA [35, 36]. It was also the most commonly applied sleep-specific instrument in economic evaluations [14]. Participants rate their propensity to doze or fall asleep on a four-point scale from 0 (never) to 3 (high chance) in eight different low-activity situations. Taking the sum of each item’s score, ranging from 0 to 24, an ESS score greater than 10 is indicative of excessive daytime sleepiness [37].  The PSQI is a self-report questionnaire used to discriminate between ‘poor’ and ‘good’ sleepers in clinical and non-clinical populations. Its application within sleep disorders is broad including sleep initiation and maintenance disorders and disorders of excessive somnolence [38]. Nineteen items generate 7 component scores (based on Likert response scored 0 to 3) and an overall ‘global’ score ranging from 0 to 21. Five additional items answered by a bed partner are excluded from scoring.  Higher scores for the EQ-5D-5L, ICECAP-A and FOSQ-10 indicated better outcomes, whereas the converse was true for the ESS and the PSQI.  The EQ-5D-5L is a widely-used generic health-related QoL measure. Although not validated in sleep disordered populations, the EQ-5D-5L has shown excellent psychometric properties across a range of other disease populations and settings [26]. The EQ-5D, both 3- and 5- level, are the most frequently used generic instrument in economic evaluations within sleep disorders [14], though the EQ-5D-5L has reduced ceiling effects compared to its three-level counterpart [27]. It measures five dimensions: mobility, self-care, usual activities, pain/discomfort, and anxiety/depression each with five levels of impairment ranging from ‘no problem’ to ‘unable’ defining a possible 3125 health states. Using the Australian general population preference weights, a utility score ranging from -0.30 to 1 was attached to each health state [28].  The ICECAP-A is a generic measure of adult capability wellbeing measuring five dimensions: stability, attachment, achievement, autonomy, and enjoyment. Each attribute has four levels ranging from full to no capability to distinguish a possible 1024 capability states [15]. The algorithm based on a best-worst scaling method using UK population tariffs was applied to score the ICECAP-A and derive overall scores ranging from 0 (no capability) to 1 (full capability) [29].  The FOSQ-10 is a self-administered questionnaire assessing the impact of excessive somnolence on QoL in adults that is often applied in primary sleep disorders, particularly OSA. Shorter than its predecessor, the FOSQ-30 [30], the FOSQ-10 has performed well in recent studies [31, 32] and a newly established minimally important difference [33] improves its interpretability and applicability in practice. Due to the limited number of items per subscale (1 to 3), only the total score (ranging from 5 to 20) is recommended for use [34].  Applied across many sleep disorders and settings, the ESS is the most commonly used instrument for assessing excessive daytime sleepiness, which is a major consideration specifically in clinical decision-making concerning the diagnosis and management of OSA [35, 36]. It was also the most commonly applied sleep-specific instrument in economic evaluations [14]. Participants rate their propensity to doze or fall asleep on a four-point scale from 0 (never) to 3 (high chance) in eight different low-activity situations. Taking the sum of each item’s score, ranging from 0 to 24, an ESS score greater than 10 is indicative of excessive daytime sleepiness [37].  The PSQI is a self-report questionnaire used to discriminate between ‘poor’ and ‘good’ sleepers in clinical and non-clinical populations. Its application within sleep disorders is broad including sleep initiation and maintenance disorders and disorders of excessive somnolence [38]. Nineteen items generate 7 component scores (based on Likert response scored 0 to 3) and an overall ‘global’ score ranging from 0 to 21. Five additional items answered by a bed partner are excluded from scoring.  Higher scores for the EQ-5D-5L, ICECAP-A and FOSQ-10 indicated better outcomes, whereas the converse was true for the ESS and the PSQI.” |
| Bias | 9 | Describe any efforts to address potential sources of bias | 5-6,8 | “…data from adults (aged 18 and above) who self-reported being diagnosed with a sleep disorder (participants were asked to specify which). Eligibility was based on predetermined quotas for various traits such as age, sleep disorder history, ethnicity, income, education, and employment status. Authentication criteria were applied to ensure legitimacy of respondents including CAPTCHA (Completely Automated Public Turing test to tell Computers and Humans Apart) test, open ends connectivity, internet protocol (IP) geolocation, and speeder limits. The goal was to secure a nationally representative sample of the Australian population. The target sample size was 1,500, well exceeding the minimum recommended number of 50 participants for this kind of survey [25].  … Four sensitivity analyses, testing the results’ robustness, compared correlation matrices used (polychoric vs Pearson’s), factor extraction method (iterative principal factor vs maximum likelihood), alternative rotation method (promax vs oblimin), and outliers (using Mahalanobis distance (*D*^2^) [50]. There was no missing data to account for in our analyses” |
| Study size | 10 | Explain how the study size was arrived at | 6 | “The target sample size was 1,500, well exceeding the minimum recommended number of 50 participants for this kind of survey [25].” |

| Quantitative variables | 11 | Explain how quantitative variables were handled in the analyses. If applicable, describe which groupings were chosen and why | 7 | “Descriptive statistics (means, standard deviations, medians, interquartile ranges, frequencies) were generated. The distribution of the dimension/total scores of all QoL instruments were tested for normality using the Shapiro-Francia test [39]. All instrument dimension/total scores followed non-normal distribution (Shapiro-Francia test, p<0.05), therefore, non-parametric tests of differences were applied (Wilcoxon-Mann Whitney, Kruskal Wallis, and Spearman’s correlation). Differences in dimension/total scores based on demographics (age band, gender, living arrangements, educational attainment) and self-rated QoL were tested using the Kruskal Wallis test.” |
| --- | --- | --- | --- | --- |
| Statistical methods | 12 | (a) Describe all statistical methods, including those used to control for confounding | 7-8 | “Convergent validity between dimension/total scores of all instruments was assessed using Kendall’s Tau-B rank correlation significant at the 5% level, recommended for ordinal categorical data [40]. Correlations below 0.30 were considered weak, those between 0.30 and 0.50 moderate, and those above 0.50 strong. Strong absolute correlations between instruments, indicative of convergent validity, suggested they were measuring similar constructs [40]. We hypothesised that strong convergent validity would exist between items or dimensions belonging to the same ICF domains. Divergent validity was also assessed using Kendall’s Tau-B correlation. We hypothesised that divergent validity would be confirmed if items/dimensions belonging to different ICF domains had lower or no statistically significant correlation than those belonging to the same ICF chapter.  Exploratory Factor Analysis (EFA), incorporating Polychoric correlation matrices and iterative principal factor extraction to accommodate the ordinal and multivariate non-normal nature of the data (Mardia’s kurtosis p<0.001 [41]), was conducted. Minimum average partials (MAP) [42] and Horn’s parallel analysis (PA) [43] were used to determine the optimal number of factors to retain. Promax oblique rotation with a kappa value of 3 was used to ensure realistic and statistically sound factor structures [44, 45]. The threshold for salient loadings was set at 0.32 as recommended in the literature [46]. The magnitude of loadings was interpreted as follows: ≥0.32 to <0.45 was poor, ≥0.45 to <0.55 was fair, ≥0.55 to <0.63 was good, ≥0.63 to <0.70 was very good, and ≥0.70 was excellent [47]. All candidate models were judged on their interpretability and theoretical sense to identify the most acceptable solution [48, 49]. Four sensitivity analyses testing the results’ robustness compared correlation matrices used (polychoric vs Pearson’s), factor extraction methods (iterative principal factor vs maximum likelihood), alternative rotation methods (promax vs oblimin), and outliers (using Mahalanobis distance (D^2^) [50].  There was no missing data to account for in our analyses. The assumed threshold for statistical significance in all analyses was 5% [51], and Stata version 15.1 (StataCorp, TX) was used to conduct all analyses.” |
|  |  | (b) Describe any methods used to examine subgroups and interactions | N/A | Subgroups and interactions were not analysed in this study, rather the data for the whole sample was analysed. |
|  |  | (c) Explain how missing data were addressed | 8 | “There was no missing data to account for in our analyses.” |
|  |  | (d) ~~Cohort study—If applicable, explain how loss to follow-up was addressed~~  ~~Case-control study—If applicable, explain how matching of cases and controls was addressed~~  Cross-sectional study—If applicable, describe analytical methods taking account of sampling strategy | 5-6 | “Following advertisement of the study to PureProfile’s platform of users, informed consent was gained from all potential participants before proceeding to the questionnaire which gathered data from adults (aged 18 and above) who self-reported being diagnosed with a sleep disorder (participants were asked to specify which). Eligibility was based on predetermined quotas for various traits such as age, sleep disorder history, ethnicity, income, education, and employment status. Authentication criteria were applied to ensure legitimacy of respondents including CAPTCHA (Completely Automated Public Turing test to tell Computers and Humans Apart) test, open ends connectivity, internet protocol (IP) geolocation, and speeder limits. The goal was to secure a nationally representative sample of the Australian population. The target sample size was 1,500, well exceeding the minimum recommended number of 50 participants for this kind of survey [25].” |
|  |  | (e) Describe any sensitivity analyses | 8 | “Four sensitivity analyses testing the results’ robustness compared correlation matrices used (polychoric vs Pearson’s), factor extraction methods (iterative principal factor vs maximum likelihood), alternative rotation methods (promax vs oblimin), and outliers (using Mahalanobis distance (D^2^) [50].” |
| Results | | | | |
| Participants | 13* | (a) Report numbers of individuals at each stage of study—eg numbers potentially eligible, examined for eligibility, confirmed eligible, included in the study, completing follow-up, and analysed | 9, Fig1 | “Of the initial PureProfile panellist population (n=5,666), 1,737 were eligible. The primary reason for exclusion was not having had a sleep disorder (n=3,488). After pre-determined quotas were reached, 1,509 eligible panellists completed the survey. There were no partial completions or missing data.” |
|  |  | (b) Give reasons for non-participation at each stage | 8, Fig1 | “The primary reason for exclusion was not having had a sleep disorder (n=3,488).”  **Exclusions (n=3,929)**  No sleep disorder (n=3,488)  Fail CAPTCHA test (n=309)  Bad open ends connection (n=81)  Fail GeoIP check (n=20)  Fail speeder limit (n=17)  Age <18 years (n=14) |
|  |  | (c) Consider use of a flow diagram | Fig1 | Please see Figure 1. |
| Descriptive data | 14* | (a) Give characteristics of study participants (eg demographic, clinical, social) and information on exposures and potential confounders | 9,  Table 1, Table 2 | “Participant characteristics are presented in Table 1. Participants’ ages ranged from 18 to 86 years (mean 46 and median 45). The sample consisted of nearly equal numbers of males and females. Self-rated QoL was indicated as average, good, or very good by 80.7% of participants. The frequency of self-reported sleep disorders is reported in Table 2. Bruxism was most commonly reported (n=569, 37.7%), followed by insomnia (n=553, 36.7%) and OSA (n=467, 31.0%). Narcolepsy was least frequently reported (n=41, 2.7%).” |
|  |  | (b) Indicate number of participants with missing data for each variable of interest | 9 | “There were no partial completions or missing data.” |
|  |  | (c) Cohort study—Summarise follow-up time (eg, average and total amount) | N/A | This is a cross-sectional study design. |
| Outcome data | 15* | Cohort study—Report numbers of outcome events or summary measures over time | N/A | This is a cross-sectional study design. |
|  |  | Case-control study—Report numbers in each exposure category, or summary measures of exposure | N/A | This is a cross-sectional study design. |
|  |  | Cross-sectional study—Report numbers of outcome events or summary measures | 10,  Table 1, Supplementary material | “The distribution of total (ESS, FOSQ, PSQI) and utility (EQ-5D-5L, ICECAP-A) scores across participant characteristics is shown in Table 1. Median (IQR) EQ-5D-5L and ICECAP-A utility scores were 0.89 (0.78-0.96) and 0.85 (0.69-0.94) respectively. Median (IQR) total scores on the ESS, FOSQ-10, and PSQI were 6 (3-9), 35 (28-38), and 9 (6-12), respectively. For all five instruments, there was a statistically significant relationship between utility/total score and self-rated QoL, suggesting that all instruments were able to discriminate according to self-rated QoL (Kruskal Wallis test, p<0.01). The distribution of utility/total scores for all instruments across all dimension levels of the EQ-5D-5L and ICECAP-A are presented in the supplementary material Tables S1 and S2.” |
| Main results | 16 | (a) Give unadjusted estimates and, if applicable, confounder-adjusted estimates and their precision (eg, 95% confidence interval). Make clear which confounders were adjusted for and why they were included | 9-12  Tables 1-4, Supplementary material | All results reported in-text (Results section) and Tables 1-4 are unadjusted estimates. Confounder adjustment was not performed in this study. |
|  |  | (b) Report category boundaries when continuous variables were categorized | Table 1 | Age group (years): 18-24, 25-34, 35-44, 45-54, 55-64, 65-75, 75+ |
|  |  | (c) If relevant, consider translating estimates of relative risk into absolute risk for a meaningful time period | N/A |  |

| Other analyses | 17 | Report other analyses done—eg analyses of subgroups and interactions, and sensitivity analyses | 11-12 | “Comparing polychoric and Pearson’s correlation matrices used for EFA, the determinant, Bartlett’s test of sphericity, and KMO results (Tables 4 and S6) show that either correlation matrix was similarly appropriate. Factor extraction using maximum likelihood was similarly parsimonious to iterative principal factor extraction, Factors 1 and 2 explained 48.2% and 48.5% of total variance before rotation respectively with similar uniqueness values. Promax and oblimin rotated solutions shared the same factor loadings and loading magnitudes. Based on the critical value (D^2^=59.7) with 30 degrees freedom and using the conservative p<0.001 significance level, 146 observations were identified as potential outliers. EFA excluding outliers shared the same close fit (RMSR=0.041) with identical factor loadings of similar magnitude (supplementary Table S7) and was also robust to extraction and rotation methods. Therefore, our EFA results were robust across correlation matrices, extraction method, rotation, and outliers.” |
| --- | --- | --- | --- | --- |
| Discussion | | | | |
| Key results | 18 | Summarise key results with reference to study objectives | 12 | “Weak to moderate convergence between the generic versus sleep-specific instruments supports their application in parallel versus as alternatives within an economic evaluation. Our results suggest that, due to their wider coverage of QoL concepts as summarised by the ICF framework, the EQ-5D-5L in conjunction with the ESS or PSQI are the most appropriate instruments amongst those used in this study to apply dependent on context, content coverage, and latent constructs in economic evaluations.” |
| Limitations | 19 | Discuss limitations of the study, taking into account sources of potential bias or imprecision. Discuss both direction and magnitude of any potential bias | 14 | “Several limitations of this study merit note. Data used in this study was collected using a cross-sectional study design where future research should consider a longitudinal study design. Such study design would allow the evaluation of which instruments are most responsive and best capture clinically important changes over time, supporting the assessment of incremental effectiveness in economic evaluation [63, 64]. Further, expanding the comparisons to include other generic and/or sleep-specific instruments should be considered because they may be more applicable in the assessment of sleep health interventions. For example the newly-developed SF-6Dv2 [65] has shown comparative validity to the EQ-5D-5L [66] but is more sensitive to clinical improvements in OSA patients [67], and a recently derived value set enables the calculation of QALYs for health technology assessment [68]. Other jurisdictions should extrapolate our results cautiously given they are based on a nationally representative sample of Australians who self-reported having a sleep disorder. As such, replication of this study in other countries is appropriate.” |
| Interpretation | 20 | Give a cautious overall interpretation of results considering objectives, limitations, multiplicity of analyses, results from similar studies, and other relevant evidence | 13, 14 | “In the absence of a ‘gold standard’ instrument capturing relevant attributes of QoL in sleep disorders, which combination of instruments to use within economic evaluations in general sleep disorder populations should be determined based on study aim(s), content coverage, and latent constructs. The economic evaluation results should enable standardised comparisons across other non-sleep disorders, as in a CUA or CEA. In the case where utility scores are required, as in a CUA, the EQ-5D-5L is one of the instruments recommended by decision-making bodies such as NICE and MSAC [9, 10]. While the EQ-5D-5L remains the most applied instrument in economic evaluations of sleep interventions, the ICECAP-A would be more appropriate in contexts where there is a need to reflect changes in wellbeing-related capability. However, some measurement properties of generic QoL instruments in sleep disorder populations have yet to be demonstrated. Therefore, using a complementary sleep-specific instrument in parallel is required, and ICF framework coverage and latent constructs are additional considerations informing instrument choice. The ICF ‘activities and participation’ domain was captured more than the ‘body functions’ domain. Between the generic EQ-5D-5L and ICECAP-A, the former covers more of the ICF framework (measuring two ICF domains) and two latent constructs (Factors 3 [‘wellbeing’] and 4 [‘physical health’]), suggestive of broader coverage of QoL aspects. Of the sleep-specific instruments, the ICF framework was most broadly covered by the ESS, but the PSQI better measured the ‘body functions’ domain. The consideration of latent constructs shows that ‘daytime dysfunction’ (Factor 1) is common to all sleep-specific instruments, however the ESS and PSQI measure one additional factor each, ‘fatigue’ (Factor 2) and ‘perceived sleep quality’ (Factor 5) respectively. Within sleep disorder cohorts in general, the combination of generic and sleep-specific instrument should be guided by the context in which they will be applied. From the selection of instruments used in this study, a full economic evaluation should employ the EQ-5D-5L as the generic instrument to allow QALY calculation. The accompanying sleep-specific instrument depends on aspects of QoL that need to be measured, with the ESS offering the broadest content coverage and measurement of latent constructs. Although, the PSQI would be more appropriate in contexts where measuring ‘body functions’ is of import.” |
| Generalisability | 21 | Discuss the generalisability (external validity) of the study results | 14 | “Other jurisdictions should extrapolate our results cautiously given they are based on a nationally representative sample of Australians who self-reported having a sleep disorder. As such, replication of this study in other countries is appropriate.” |
| Other information | |  | | |
| Funding | 22 | Give the source of funding and the role of the funders for the present study and, if applicable, for the original study on which the present article is based | 21 | “The authors declare that no funds, grants, or other support were received during the preparation of this manuscript.” |

*Give information separately for cases and controls in case-control studies and, if applicable, for exposed and unexposed groups in cohort and cross-sectional studies.

**Note:** An Explanation and Elaboration article discusses each checklist item and gives methodological background and published examples of transparent reporting. The STROBE checklist is best used in conjunction with this article (freely available on the Web sites of PLoS Medicine at http://www.plosmedicine.org/, Annals of Internal Medicine at http://www.annals.org/, and Epidemiology at http://www.epidem.com/). Information on the STROBE Initiative is available at www.strobe-statement.org.
