# Supplementary material for: An empirical comparison of sleep-specific versus generic quality of life instruments among Australians with sleep disorders
Source: Qual Life Res. 2024 Jun 24;33(8):2261–74. doi: 10.1007/s11136-024-03686-0 (PMC11286652; doi:10.1007/s11136-024-03686-0)
Supplement: Supplementary file 2 — Supplementary file2 (DOCX 52 KB) [file 11136_2024_3686_MOESM2_ESM.docx]

**AN EMPIRICAL COMPARISON OF SLEEP-SPECIFIC VERSUS GENERIC QUALITY OF LIFE INSTRUMENTS AMONG AUSTRALIANS WITH SLEEP DISORDERS**

**Quality of Life Research**

Taylor-Jade Woods^1^*, Billingsley Kaambwa^1^

^1^ Health Economics Unit, College of Medicine and Public Health, Flinders University, Bedford Park, South Australia, Australia

*Corresponding author

*Taylor-Jade Woods, BMedSc(Hons), Health Economics Unit, College of Medicine and Public Health, Flinders University, Adelaide, Australia

E: [taylorjade.woods@flinders.edu.au](mailto:taylorjade.woods@flinders.edu.au)

ORCID ID: 0000-0002-0545-0401

Billingsley Kaambwa, PhD (Health Economics), College of Medicine and Public Health, Flinders University, Adelaide, Australia

E: [billingsley.kaambwa@flinders.edu.au](mailto:billingsley.kaambwa@flinders.edu.au)

ORCID ID: 0000-0002-2128-3404

Table S1. Distribution of ICECAP-A, ESS, FOSQ-10, and PSQI total scores across dimensions of the EQ-5D-5L.

| EQ-5D-5L  dimensions and levels | Frequency  n (%) | Median ICECAP-A | Median  ESS | Median FOSQ-10 | Median  PSQI |
| --- | --- | --- | --- | --- | --- |
| Mobility |  |  |  |  |  |
| I have no problems in walking about | 1,012 (67.1) | 0.88 | 5 | 36 | 9 |
| I have slight problems in walking about | 291 (19.3) | 0.79 | 7 | 31 | 11 |
| I have moderate problems in walking about | 144 (9.5) | 0.74 | 9 | 30 | 12 |
| I have severe problems in walking about | 55 (3.6) | 0.50 | 9 | 29 | 13 |
| I am unable to walk about | 7 (0.5) | 0.53 | 7 | 33 | 11 |
| Self-care |  |  |  |  |  |
| I have no problems washing or dressing myself | 1,247 (82.6) | 0.88 | 5 | 36 | 9 |
| I have slight problems washing or dressing myself | 142 (9.4) | 0.71 | 8 | 28 | 12 |
| I have moderate problems washing or dressing myself | 95 (6.3) | 0.64 | 11 | 23 | 12 |
| I have severe problems washing or dressing myself | 20 (1.3) | 0.73 | 10 | 24 | 12 |
| I am unable to wash or dress myself | 5 (0.3) | 0.53 | 9 | 24 | 11 |
| Usual activities |  |  |  |  |  |
| I have no problems doing my usual activities | 955 (63.3) | 0.90 | 5 | 26 | 9 |
| I have slight problems doing my usual activities | 311 (20.6) | 0.79 | 6 | 32 | 11 |
| I have moderate problems doing my usual activities | 176 (11.7) | 0.67 | 9 | 28 | 12 |
| I have severe problems doing my usual activities | 57 (3.8) | 0.55 | 10 | 25 | 13 |
| I am unable to do my usual activities | 10 (0.7) | 0.36 | 10 | 24 | 11 |
| Pain/discomfort |  |  |  |  |  |
| I have no pain or discomfort | 458 (30.4) | 0.92 | 5 | 37 | 8 |
| I have slight pain or discomfort | 569 (37.7) | 0.88 | 6 | 35 | 10 |
| I have moderate pain or discomfort | 341 (22.6) | 0.78 | 6 | 32 | 11 |
| I have severe pain or discomfort | 116 (7.7) | 0.60 | 9 | 29 | 13 |
| I have extreme pain or discomfort | 25 (1.7) | 0.44 | 10 | 25 | 16 |
| Anxiety/depression |  |  |  |  |  |
| I am not anxious or depressed | 508 (33.7) | 0.93 | 5 | 38 | 8 |
| I am slightly anxious or depressed | 446 (29.6) | 0.85 | 6 | 35 | 10 |
| I am moderately anxious or depressed | 370 (24.5) | 0.74 | 7 | 31 | 11 |
| I am severely anxious or depressed | 128 (8.5) | 0.62 | 8 | 27 | 12 |
| I am extremely anxious or depressed | 57 (3.8) | 0.51 | 8 | 26 | 14 |
| EQ-5D-5L: EuroQoL 5-Dimension 5-Level, ICECAP-A: ICEpop CAPability measure for Adults, ESS: Epworth Sleepiness Scale, FOSQ-10: 10-Item Functional Outcomes of Sleep Questionnaire, PSQI: Pittsburgh Sleep Quality Index. | | | | | |

Table S2. Distribution of EQ-5D-5L, ESS, FOSQ-10, and PSQI total scores across dimensions of the ICECAP-A

| ICECAP-A dimensions and levels | Frequency  n (%) | Median  EQ-5D-5L | Median  ESS | Median FOSQ-10 | Median  PSQI |
| --- | --- | --- | --- | --- | --- |
| Stability |  |  |  |  |  |
| I am able to feel settled and secure in all areas of my life | 426 (28.2) | 0.84 | 5 | 38 | 8 |
| I am able to feel settled and secure in many areas of my life | 636 (42.2) | 0.75 | 6 | 35 | 10 |
| I am able to feel settled and secure in few areas of my life | 356 (23.6) | 0.65 | 7 | 31 | 12 |
| I am unable to feel settled and secure in any areas of my life | 91 (6.0) | 0.39 | 7 | 29 | 12 |
| Attachment |  |  |  |  |  |
| I can have a lot of love, friendship, and support | 517 (34.3) | 0.84 | 5 | 37 | 9 |
| I can have quite a lot of love, friendship, and support | 579 (38.4) | 0.75 | 6 | 35 | 10 |
| I can have a little of love, friendship, and support | 355 (23.5) | 0.64 | 7 | 31 | 11 |
| I cannot have any love, friendship, and support | 58 (3.8) | 0.41 | 7 | 29 | 14 |
| Autonomy |  |  |  |  |  |
| I am able to be completely independent | 846 (56.1) | 0.80 | 5 | 37 | 9 |
| I am able to be independent in many things | 506 (33.5) | 0.68 | 7 | 32 | 11 |
| I am able to be independent in a few things | 141 (9.3) | 0.44 | 9 | 26 | 12 |
| I am unable to be at all independent | 16 (1.1) | 0.18 | 9 | 22 | 13 |
| Achievement |  |  |  |  |  |
| I can achieve and progress in all aspects of my life | 464 (30.8) | 0.84 | 5 | 38 | 8 |
| I can achieve and progress in many aspects of my life | 689 (45.7) | 0.75 | 6 | 35 | 10 |
| I can achieve and progress in few aspects of my life | 293 (19.4) | 0.62 | 7 | 30 | 12 |
| I cannot achieve and progress in any aspects of my life | 63 (4.2) | 0.29 | 8 | 27 | 15 |
| Enjoyment |  |  |  |  |  |
| I can have a lot of enjoyment and pleasure | 452 (30.0) | 0.84 | 5 | 37 | 8 |
| I can have quite a lot of enjoyment and pleasure | 665 (44.1) | 0.75 | 6 | 35 | 10 |
| I can have a little enjoyment and pleasure | 360 (23.9) | 0.62 | 7 | 31 | 12 |
| I cannot have any enjoyment and pleasure | 32 (2.1) | 0.32 | 7 | 26 | 16 |
| EQ-5D-5L: EuroQoL 5-Dimension 5-Level, ICECAP-A: ICEpop CAPability measure for Adults, ESS: Epworth Sleepiness Scale, FOSQ-10: 10-Item Functional Outcomes of Sleep Questionnaire, PSQI: Pittsburgh Sleep Quality Index. | | | | | |

Table s3. Classification of the EQ-5D-5L, ICECAP-A, ESS, FOSQ-10, and PSQI items/dimensions according to the International Classification of Functioning and Disability Functioning (ICF) framework.

| **International Classification of Functioning, Disability and Health (ICF) Classifications** | | | **Quality of life instruments** | | | | |
| --- | --- | --- | --- | --- | --- | --- | --- |
| **ICF Domain** | **ICF Chapters** | **ICF Chapter & Categories** | **EQ-5D-5L**  **5 dimensions** | **ICECAP-A**  **5 dimensions** | **ESS**  **8 items** | **FOSQ-10**  **10 items** | **PSQI**  **7 dimensions** |
| Body Function | Mental functions  Sensory functions and pain  Voice and speech functions  Functions of the cardiovascular, haematological, immunological and respiratory systems  Functions of the digestive, metabolic and endocrine systems  Genitourinary and reproductive functions  Neuromusculoskeletal and movement-related functions  Functions of the skin and related structures | *Chapter b1: Mental Functions*   - b126 temperament and personality functions - b130 energy and drive functions - b134 sleep functions - b140 attention functions - b144 memory functions - b152 emotional functions | Anxiety/ depression | N/A | Sitting and reading  Watching TV  Sitting, inactive  Car passenger  Lying down to rest  Sitting and talking  Sitting quietly  In a car | Concentration  Remembering  Finishing a meal  Working on a hobby  Housework  Operating motor vehicle (short)  Operating motor vehicle (long)  Difficulty completing tasks  Managing financial affairs  Performing work | Sleep quality  Sleep latency  Sleep duration  Sleep efficiency  Sleep disturbances  Daytime dysfunction |
|  |  | *Chapter b2: Sensory functions and pain*   - b280 sensation of pain - b298 sensation of pain, other specified - b299 sensation of pain, unspecified | Pain/ discomfort | N/A | N/A | N/A | Sleep disturbances |
| Activities and Participation | Learning and applying knowledge  General tasks and demands  Communication  Mobility  Self-care  Domestic life  Interpersonal interactions and relationships  Major life areas  Community, social and civic life | *Chapter d1: Learning and applying knowledge*   - d110 watching - b115 listening | N/A | Achievement | Watching TV | N/A | N/A |
|  |  | *Chapter d2: General tasks and demands*   - d210 undertaking a single task - d220 undertaking multiple tasks - d230 carrying out daily routine - d298 general tasks and demands, other specified - d299 general tasks and demands, unspecified | Usual activities | Security  Autonomy  Achievement | In a car | Working on a hobby  Housework  Operating motor vehicle (short)  Operating motor vehicle (long)  Difficulty completing tasks  Performing work | Daytime dysfunction |
|  |  | *Chapter d3: Communication*   - d310 communicating with – receiving – spoken messages - d315 communicating with – receiving – non-verbal messages - d330 speaking - d335 producing non-verbal messages - d350 conversation - d355 discussion | N/A | N/A | Sitting and talking | N/A | N/A |
|  |  | *Chapter d4: Mobility*   - d410 changing basic body position - d415 maintaining body position - d420 transferring oneself - d429 changing and maintaining body position, other specified and unspecified - d430 lifting and carrying objects - d435 moving objects with lower extremities - d445 hand and arm use - d446 fine foot use - d450 walking - d455 moving around - d460 moving around in different locations - d470 using transportation - d475 driving | Mobility | N/A | Sitting and reading  Car passenger  In a car | Operating motor vehicle (short)  Operating motor vehicle (long)  Difficulty completing tasks | Daytime dysfunction |
|  |  | *Chapter d5: Self-care*   - d510 washing oneself - d520 caring for body parts - d530 toileting - d540 dressing - d550 eating - d560 drinking - d570 looking after one’s health - d598 self-care, other specified - d599 self-care, - unspecified | Self-care | Autonomy | Lying down to rest | Finishing a meal | Use of sleep medication |
|  |  | *Chapter d6: Domestic life*   - d620 acquisition of goods and services - d629 acquisition of goods and services, other specified and unspecified - d630-d649 household tasks | Usual activities | Security  Autonomy | N/A | Housework | N/A |
|  |  | *Chapter d7: Interpersonal interactions and relationships*   - d710-d729 general interpersonal interactions - d740 formal relationships - d750 informal social relationships - d760 family relationships - d779 particular interpersonal relationships, other specified and unspecified - d798 Interpersonal interactions and relationships, other specified - d799 Interpersonal interactions and relationships, unspecified | Usual activities | Autonomy  Attachment | Sitting and talking | N/A | Daytime dysfunction |
|  |  | *Chapter d8: Major life areas*   - d840 apprenticeship (work preparation) - d845 acquiring, keeping and terminating a job - d850 remunerative employment - d855 non-remunerative employment - d859 work and employment, other specified and unspecified - d860 basic economic transactions - d865 complex economic transactions - d870 economic self-sufficiency - d879 economic life, other specified and unspecified | Usual activities | Achievement | N/A | Managing financial affairs  Performing work | N/A |
|  |  | *Chapter d9: Community, social and civic life*   - d910 community life - d920 recreation and leisure - d930 religion and spirituality - d998 community, social and civic life, other specified - d999 community, social and civic life, unspecified | Usual activities | Enjoyment | Sitting and reading  Sitting and talking  Watching TV  Sitting, inactive  Sitting quietly | Working on a hobby | Daytime dysfunction |
| Abbreviations: EQ-5D-5L: EuroQoL 5-Dimension 5-Level, ICECAP-A: ICEpop CAPability measure for Adults, ESS: Epworth Sleepiness Scale, FOSQ-10: 10-item Functional Outcomes of Sleep Questionnaire, PSQI: Pittsburgh Sleep Quality Index. | | | | | | | |

Table S4. Kendall’s Tau-B rank correlation coefficients showing the correlation between the item or dimension scores of the EuroQoL 5-dimension 5-level (EQ-5D-5L), ICEpop CAPability measure for Adults (ICECAP-A), Epworth Sleepiness Scale (ESS), 10-item Functional Outcomes of Sleep Questionnaire (FOSQ-10), and Pittsburgh Sleep Quality Index (PSQI).

|  | EQ-5D-5L | | | | | ICECAP-A | | | | |
| --- | --- | --- | --- | --- | --- | --- | --- | --- | --- | --- |
|  | Mobility | Self care | Usual Activities | Pain | Anxiety/  Depression | Stability | Attachment | Autonomy | Achievement | Enjoyment |
| EQ-5D-5L |  |  |  |  |  |  |  |  |  |  |
| Mobility | - |  |  |  |  |  |  |  |  |  |
| Self Care | **0.51** | - |  |  |  |  |  |  |  |  |
| Usual activities | **0.62** | **0.57** | - |  |  |  |  |  |  |  |
| Pain | **0.46** | **0.34** | **0.49** | - |  |  |  |  |  |  |
| Anxiety/Depression | **0.16** | **0.23** | **0.29** | **0.28** | - |  |  |  |  |  |
| ICECAP-A |  |  |  |  |  |  |  |  |  |  |
| Stability | **0.20** | **0.19** | **0.31** | **0.30** | **0.47** | - |  |  |  |  |
| Attachment | **0.16** | **0.17** | **0.24** | **0.27** | **0.34** | **0.50** | - |  |  |  |
| Autonomy | **0.36** | **0.41** | **0.45** | **0.34** | **0.29** | **0.35** | **0.27** | - |  |  |
| Achievement | **0.29** | **0.28** | **0.41** | **0.37** | **0.33** | **0.54** | **0.48** | **0.45** | - |  |
| Enjoyment | **0.24** | **0.22** | **0.32** | **0.34** | **0.38** | **0.57** | **0.55** | **0.36** | **0.58** | - |
| ESS |  |  |  |  |  |  |  |  |  |  |
| Sitting and reading | **0.15** | **0.14** | **0.18** | **0.12** | **0.15** | **0.13** | **0.12** | **0.16** | **0.15** | **0.11** |
| Watching TV | **0.10** | **0.11** | **0.12** | **0.10** | **0.14** | **0.12** | **0.11** | **0.13** | **0.10** | **0.09** |
| Sitting, inactive | **0.14** | **0.21** | **0.15** | **0.08** | **0.09** | 0.04 | **0.06** | **0.13** | **0.07** | 0.03 |
| Car passenger | **0.08** | **0.14** | **0.15** | **0.06** | **0.13** | **0.10** | **0.06** | **0.15** | **0.10** | 0.04 |
| Lying down to rest | **0.11** | **0.07** | **0.13** | **0.12** | **0.13** | **0.16** | **0.11** | **0.13** | **0.14** | **0.11** |
| Sitting and talking | **0.18** | **0.31** | **0.19** | **0.07** | **0.12** | 0.03 | 0.04 | **0.17** | 0.05 | 0.02 |
| Sitting quietly | **0.15** | **0.24** | **0.20** | **0.12** | **0.14** | **0.10** | **0.08** | **0.19** | **0.12** | **0.10** |
| In a car | **0.13** | **0.27** | **0.15** | 0.04 | **0.08** | -0.02 | -0.01 | **0.15** | 0.04 | -0.02 |
| PSQI |  |  |  |  |  |  |  |  |  |  |
| Sleep Quality | **0.15** | **0.10** | **0.19** | **0.26** | **0.30** | **0.32** | **0.24** | **0.19** | **0.26** | **0.33** |
| Sleep Latency | **0.08** | **0.08** | **0.14** | **0.16** | **0.27** | **0.24** | **0.19** | **0.13** | **0.22** | **0.23** |
| Sleep Duration | **0.06** | **0.06** | **0.05** | **0.11** | **0.08** | **0.12** | **0.09** | **0.05** | **0.13** | **0.16** |
| Sleep Efficiency | **0.11** | **0.09** | **0.11** | **0.13** | **0.09** | **0.09** | **0.08** | **0.08** | **0.14** | **0.15** |
| Sleep Disturbances | **0.22** | **0.18** | **0.25** | **0.32** | **0.28** | **0.27** | **0.18** | **0.23** | **0.28** | **0.25** |
| Use of Sleep Medications | **0.18** | **0.18** | **0.19** | **0.18** | **0.13** | **0.08** | **0.07** | **0.19** | **0.11** | **0.11** |
| Daytime Dysfunction | **0.17** | **0.25** | **0.27** | **0.20** | **0.35** | **0.29** | **0.21** | **0.28** | **0.29** | **0.25** |
| FOSQ-10 |  |  |  |  |  |  |  |  |  |  |
| Concentration | **0.15** | **0.23** | **0.26** | **0.23** | **0.34** | **0.29** | **0.20** | **0.29** | **0.27** | **0.24** |
| Remembering | **0.16** | **0.23** | **0.28** | **0.22** | **0.34** | **0.28** | **0.18** | **0.27** | **0.25** | **0.24** |
| Finishing a meal | **0.20** | **0.32** | **0.26** | **0.13** | **0.23** | **0.10** | **0.08** | **0.27** | **0.12** | **0.09** |
| Working on a hobby | **0.18** | **0.25** | **0.29** | **0.22** | **0.31** | **0.28** | **0.18** | **0.27** | **0.26** | **0.23** |
| Housework | **0.23** | **0.26** | **0.33** | **0.26** | **0.34** | **0.33** | **0.22** | **0.32** | **0.29** | **0.28** |
| Operating motor vehicle (short) | **0.18** | **0.32** | **0.26** | **0.16** | **0.21** | **0.10** | **0.09** | **0.25** | **0.14** | **0.13** |
| Operating motor vehicle (long) | **0.19** | **0.30** | **0.25** | **0.16** | **0.21** | **0.14** | **0.12** | **0.23** | **0.18** | **0.16** |
| Difficulty completing tasks | **0.20** | **0.33** | **0.32** | **0.20** | **0.30** | **0.24** | **0.17** | **0.30** | **0.25** | **0.19** |
| Managing financial affairs | **0.21** | **0.31** | **0.26** | **0.17** | **0.28** | **0.21** | **0.15** | **0.29** | **0.21** | **0.16** |
| Performing work | **0.22** | **0.34** | **0.33** | **0.20** | **0.32** | **0.25** | **0.17** | **0.31** | **0.24** | **0.21** |

|  | ESS | | | | | | | | PSQI | | | | | | |
| --- | --- | --- | --- | --- | --- | --- | --- | --- | --- | --- | --- | --- | --- | --- | --- |
|  | Sitting and reading | Watching TV | Sitting, inactive | Car passen-ger | Lying down to rest | Sitting and talking | Sitting quietly | In a car | Sleep quality | Sleep latency | Sleep duration | Sleep efficiency | Sleep disturbances | Use of sleep medication | Daytime dys-function |
| ESS |  |  |  |  |  |  |  |  |  |  |  |  |  |  |  |
| Sitting and reading | - |  |  |  |  |  |  |  |  |  |  |  |  |  |  |
| Watching TV | **0.50** | - |  |  |  |  |  |  |  |  |  |  |  |  |  |
| Sitting, inactive | **0.43** | **0.42** | - |  |  |  |  |  |  |  |  |  |  |  |  |
| Car passenger | **0.43** | **0.39** | **0.50** | - |  |  |  |  |  |  |  |  |  |  |  |
| Lying down to rest | **0.36** | **0.35** | **0.28** | **0.37** | - |  |  |  |  |  |  |  |  |  |  |
| Sitting and talking | **0.36** | **0.28** | **0.56** | **0.41** | **0.16** | - |  |  |  |  |  |  |  |  |  |
| Sitting quietly | **0.44** | **0.39** | **0.51** | **0.45** | **0.35** | **0.50** | - |  |  |  |  |  |  |  |  |
| In a car | **0.31** | **0.26** | **0.51** | **0.43** | **0.15** | **0.65** | **0.49** | - |  |  |  |  |  |  |  |
| PSQI |  |  |  |  |  |  |  |  |  |  |  |  |  |  |  |
| Sleep quality | **0.13** | **0.12** | **0.07** | **0.10** | **0.11** | 0.03 | **0.12** | 0.02 | - |  |  |  |  |  |  |
| Sleep latency | 0.04 | 0.04 | 0.02 | **0.06** | 0.04 | -0.01 | **0.06** | 0.00 | **0.41** | - |  |  |  |  |  |
| Sleep duration | **0.07** | **0.08** | 0.05 | 0.04 | 0.03 | -0.02 | 0.05 | 0.00 | **0.40** | **0.27** | - |  |  |  |  |
| Sleep efficiency | **0.06** | 0.02 | 0.02 | 0.03 | 0.02 | -0.02 | 0.04 | **-0.02** | **0.28** | **0.26** | **0.47** | - |  |  |  |
| Sleep disturbances | **0.22** | **0.18** | **0.21** | **0.20** | **0.19** | **0.16** | **0.20** | **0.13** | **0.40** | **0.34** | **0.20** | **0.20** | - |  |  |
| Use of sleep medication | **0.09** | **0.07** | **0.16** | **0.09** | -0.03 | **0.19** | **0.14** | **0.17** | **0.13** | **0.17** | **0.06** | **0.11** | **0.25** | - |  |
| Daytime dysfunction | **0.28** | **0.22** | **0.30** | **0.30** | **0.21** | **0.30** | **0.32** | **0.32** | **0.32** | **0.22** | **0.15** | **0.13** | **0.38** | **0.27** | - |
| FOSQ-10 |  |  |  |  |  |  |  |  |  |  |  |  |  |  |  |
| Concentration | **0.28** | **0.26** | **0.28** | **0.29** | **0.25** | **0.28** | **0.32** | **0.28** | **0.28** | **0.21** | **0.13** | **0.09** | **0.31** | **0.16** | **0.46** |
| Remembering | **0.27** | **0.23** | **0.27** | **0.26** | **0.20** | **0.27** | **0.30** | **0.25** | **0.26** | **0.22** | **0.14** | **0.11** | **0.32** | **0.20** | **0.45** |
| Finishing a meal | **0.24** | **0.19** | **0.37** | **0.28** | **0.09** | **0.45** | **0.35** | **0.44** | **0.08** | **0.11** | **0.06** | **0.05** | **0.25** | **0.24** | **0.39** |
| Working on a hobby | **0.28** | **0.25** | **0.30** | **0.31** | **0.24** | **0.31** | **0.33** | **0.30** | **0.22** | **0.21** | **0.12** | **0.09** | **0.30** | **0.17** | **0.47** |
| Housework | **0.25** | **0.20** | **0.25** | **0.27** | **0.25** | **0.29** | **0.32** | **0.27** | **0.26** | **0.23** | **0.11** | **0.12** | **0.34** | **0.20** | **0.46** |
| Operating motor vehicle (short) | **0.24** | **0.20** | **0.36** | **0.29** | **0.12** | **0.43** | **0.33** | **0.45** | **0.12** | **0.10** | 0.05 | 0.04 | **0.23** | **0.27** | **0.38** |
| Operating motor vehicle (long) | **0.24** | **0.22** | **0.35** | **0.31** | **0.18** | **0.35** | **0.34** | **0.38** | **0.14** | **0.10** | **0.06** | 0.05 | **0.23** | **0.23** | **0.36** |
| Difficulty completing tasks | **0.26** | **0.24** | **0.33** | **0.33** | **0.19** | **0.37** | **0.35** | **0.36** | **0.17** | **0.17** | **0.07** | 0.05 | **0.31** | **0.22** | **0.45** |
| Managing financial affairs | **0.25** | **0.21** | **0.33** | **0.28** | **0.15** | **0.39** | **0.30** | **0.38** | **0.13** | **0.13** | **0.05** | 0.03 | **0.26** | **0.22** | **0.43** |
| Performing work | **0.26** | **0.22** | **0.35** | **0.33** | **0.21** | **0.39** | **0.35** | **0.36** | **0.18** | **0.17** | **0.07** | **0.07** | **0.30** | **0.23** | **0.47** |

|  | FOSQ-10 | | | | | | | | | |
| --- | --- | --- | --- | --- | --- | --- | --- | --- | --- | --- |
|  | Concentration | Remembering | Finishing a meal | Working on a hobby | Housework | Operating motor vehicle (short) | Operating motor vehicle (long) | Difficulty completing tasks | Managing financial affairs | Performing work |
| FOSQ-10 |  |  |  |  |  |  |  |  |  |  |
| Concentration | - |  |  |  |  |  |  |  |  |  |
| Remembering | **0.69** | - |  |  |  |  |  |  |  |  |
| Finishing a meal | **0.45** | **0.46** | - |  |  |  |  |  |  |  |
| Working on a hobby | **0.57** | **0.55** | **0.51** | - |  |  |  |  |  |  |
| Housework | **0.55** | **0.54** | **0.44** | **0.63** | - |  |  |  |  |  |
| Operating motor vehicle (short) | **0.44** | **0.45** | **0.59** | **0.48** | **0.44** | - |  |  |  |  |
| Operating motor vehicle (long) | **0.43** | **0.42** | **0.48** | **0.45** | **0.44** | **0.72** | - |  |  |  |
| Difficulty completing tasks | **0.52** | **0.51** | **0.52** | **0.57** | **0.56** | **0.60** | **0.59** | - |  |  |
| Managing financial affairs | **0.48** | **0.47** | **0.57** | **0.54** | **0.51** | **0.56** | **0.52** | **0.61** | - |  |
| Performing work | **0.51** | **0.50** | **0.53** | **0.56** | **0.57** | **0.58** | **0.53** | **0.62** | **0.64** | - |
| **Bolded** coefficients were statistically significant (p<0.05).  Underlined coefficients indicate those which were not hypothesised to belong to the same International Classification of Functioning and Disability Functioning (ICF) domain.  Note that dimension scores for the FOSQ-10 are ‘reverse-scored’ so that a higher (lower) score implies lower (higher) health-related quality of life.  Abbreviations: EQ-5D-5L: EuroQoL 5-Dimension 5-Level, ICECAP-A: ICEpop CAPability measure for Adults, ESS: Epworth Sleepiness Scale, FOSQ: 10-item Functional Outcomes of Sleep Questionnaire, PSQI: Pittsburgh Sleep Quality Index. | | | | | | | | | | |

| **Table S5. Horn’s Parallel Analysis results for exploratory factor analysis.** | | | |
| --- | --- | --- | --- |
| **PA -- Parallel Analysis for Principal Components -- N = 1509**  **PA Eigenvalues Averaged Over 500 Replications** | | | |
|  | PCA | PA | Dif |
| 1 | 12.99518 | 1.293623 | 11.70156 |
| 2 | 4.002833 | 1.25996 | 2.742873 |
| 3 | 1.987541 | 1.233529 | 0.7540124 |
| 4 | 1.80251 | 1.211685 | 0.5908248 |
| 5 | 1.340184 | 1.19108 | 0.1491035 |
| 6 | 0.7059112 | 1.172996 | -0.4670851 |
| 7 | 0.4385277 | 1.15518 | -0.7166527 |
| 8 | 0.3208248 | 1.138284 | -0.8174591 |
| 9 | 0.2114198 | 1.122044 | -0.9106246 |
| 10 | 0.1600859 | 1.106308 | -0.9462216 |
| 11 | 0.1357551 | 1.090887 | -0.9551315 |
| 12 | 0.1229555 | 1.075747 | -0.9527918 |
| 13 | 0.0926938 | 1.06101 | -0.9683164 |
| 14 | 0.0797007 | 1.047239 | -0.9675387 |
| 15 | 0.0607481 | 1.033504 | -0.9727563 |
| 16 | 0.0369147 | 1.019476 | -0.9825613 |
| 17 | 0.0260839 | 1.006483 | -0.9803991 |
| 18 | 0.023335 | 0.9931595 | -0.9698244 |
| 19 | 0.0001202 | 0.9797679 | -0.9796477 |
| 20 | -0.0105513 | 0.9664198 | -0.9769711 |
| 21 | -0.0153781 | 0.9531842 | -0.9685624 |
| 22 | -0.0256906 | 0.9403185 | -0.9660091 |
| 23 | -0.0460715 | 0.9277847 | -0.9738562 |
| 24 | -0.0531201 | 0.9143499 | -0.96747 |
| 25 | -0.0693164 | 0.9011367 | -0.9704531 |
| 26 | -0.0762016 | 0.8876962 | -0.9638978 |
| 27 | -0.0869781 | 0.8741626 | -0.9611406 |
| 28 | -0.1029879 | 0.8605055 | -0.9634935 |
| 29 | -0.120819 | 0.845885 | -0.966704 |
| 30 | -0.138322 | 0.8317009 | -0.9700229 |
| 31 | -0.1403339 | 0.8162196 | -0.9565536 |
| 32 | -0.1779914 | 0.8006586 | -0.97865 |
| 33 | -0.1907572 | 0.783557 | -0.9743142 |
| 34 | -0.2178359 | 0.7645579 | -0.9823938 |
| 35 | -0.2368582 | 0.739899 | -0.9767572 |

**Table S6. Minimum Average Partial results for exploratory factor analysis.**

**Minimum Average Partial Correlation for Number of Principal Components**

| m | = | 0 |  | f0 | = | 0.15085682 |
| --- | --- | --- | --- | --- | --- | --- |
| m | = | 1 |  | f1 | = | 0.04796882 |
| m | = | 2 |  | f2 | = | 0.032332 |
| m | = | 3 |  | f3 | = | 0.02937295 |
| m | = | 4 |  | f4 | = | 0.02037685 |
| m | = | 5 |  | f5 | = | 0.01535909 |
| m | = | 6 |  | f6 | = | 0.01363729 |
| m | = | 7 |  | f7 | = | 0.01426042 |
| m | = | 8 |  | f8 | = | 0.01539712 |
| m | = | 9 |  | f9 | = | 0.01727243 |
| m | = | 10 |  | f10 | = | 0.01924988 |
| m | = | 11 |  | f11 | = | 0.02159205 |
| m | = | 12 |  | f12 | = | 0.02521717 |
| m | = | 13 |  | f13 | = | 0.02688692 |
| m | = | 14 |  | f14 | = | 0.0296898 |
| m | = | 15 |  | f15 | = | 0.03325024 |
| m | = | 16 |  | f16 | = | 0.03639354 |
| m | = | 17 |  | f17 | = | 0.04055236 |
| m | = | 18 |  | f18 | = | 0.04582262 |
| m | = | 19 |  | f19 | = | 0.05255821 |
| m | = | 20 |  | f20 | = | 0.05959056 |
| m | = | 21 |  | f21 | = | 0.06447587 |
| m | = | 22 |  | f22 | = | 0.07084482 |
| m | = | 23 |  | f23 | = | 0.08187381 |
| m | = | 24 |  | f24 | = | 0.08849155 |
| m | = | 25 |  | f25 | = | 0.10176235 |
| m | = | 26 |  | f26 | = | 0.11523264 |
| m | = | 27 |  | f27 | = | 0.13517567 |
| m | = | 28 |  | f28 | = | 0.1557136 |
| m | = | 29 |  | f29 | = | 0.18252677 |
| m | = | 30 |  | f30 | = | 0.22209825 |
| m | = | 31 |  | f31 | = | 0.25951417 |
| m | = | 32 |  | f32 | = | 0.34974692 |
| m | = | 33 |  | f33 | = | 0.55728115 |
| m | = | 34 |  | f34 | = | 1 |

minap procedure suggests that **6** principal components should be extracted

For comparison, the Kaiser eigenvalue > 1 rule suggests extracting **6** principal components

Table S7. Results of exploratory factor analysis based on Pearson’s correlation matrix showing factor loadings of the EQ-5D-5L, ICECAP-A and PSQI dimensions, and ESS and FOSQ-10 items.^a^

| **Variable** | **Factor 1** | **Factor 2** | **Factor 3** | **Factor 4** | **Factor 5** | **Uniqueness** |
| --- | --- | --- | --- | --- | --- | --- |
| **EQ-5D-5L Dimensions** |  |  |  |  |  |  |
| Mobility |  |  |  | 0.78 |  | 0.39 |
| Self-care |  |  |  | 0.66 |  | 0.45 |
| Usual activities |  |  |  | 0.70 |  | 0.33 |
| Pain/discomfort |  |  |  | 0.48 |  | 0.56 |
| Anxiety/depression |  |  | 0.49 |  |  | 0.60 |
| **ICECAP-A Dimensions** |  |  |  |  |  |  |
| Stability |  |  | 0.81 |  |  | 0.37 |
| Attachment |  |  | 0.69 |  |  | 0.56 |
| Autonomy |  |  | 0.36 | 0.40 |  | 0.58 |
| Achievement |  |  | 0.72 |  |  | 0.40 |
| Joy |  |  | 0.74 |  |  | 0.41 |
| **ESS Items** |  |  |  |  |  |  |
| Sitting and reading |  | 0.71 |  |  |  | 0.50 |
| Watching TV |  | 0.70 |  |  |  | 0.55 |
| Sitting, inactive |  | 0.72 |  |  |  | 0.40 |
| Car passenger |  | 0.70 |  |  |  | 0.48 |
| Lying down to rest |  | 0.56 |  |  |  | 0.68 |
| Sitting and talking |  | 0.48 |  |  |  | 0.47 |
| Sitting quietly |  | 0.67 |  |  |  | 0.45 |
| In a car |  | 0.48 |  |  |  | 0.48 |
| **FOSQ-10 Items** |  |  |  |  |  |  |
| Concentration | 0.64 |  |  |  |  | 0.41 |
| Remembering | 0.66 |  |  |  |  | 0.43 |
| Finishing a meal | 0.73 |  |  |  |  | 0.44 |
| Working on a hobby | 0.72 |  |  |  |  | 0.38 |
| Housework | 0.68 |  |  |  |  | 0.41 |
| Operating motor vehicle (short distance) | 0.83 |  |  |  |  | 0.35 |
| Operating a motor vehicle (long distance) | 0.74 |  |  |  |  | 0.46 |
| Difficulty completing tasks | 0.84 |  |  |  |  | 0.33 |
| Managing financial affairs | 0.85 |  |  |  |  | 0.36 |
| Performing work | 0.80 |  |  |  |  | 0.34 |
| **PSQI Components** |  |  |  |  |  |  |
| Sleep quality |  |  |  |  | 0.62 | 0.47 |
| Sleep latency |  |  |  |  | 0.50 | 0.66 |
| Sleep duration |  |  |  |  | 0.76 | 0.53 |
| Sleep efficiency |  |  |  |  | 0.73 | 0.58 |
| Sleep disturbances |  |  |  |  | 0.35 | 0.67 |
| Use of sleep medication |  |  |  |  |  | 0.87 |
| Daytime dysfunction | -0.41 |  |  |  |  | 0.53 |
| **Total variance explained by factor (%)** | 29.7 | 9.5 | 4.8 | 4.6 | 3.3 | n/a |
| **Determinant** | *p* < 0.001 | | | | | |
| **Bartlett’s test of sphericity** | *X*^2^ = 28151.710, *p* < 0.001 | | | | | |
| **Kaiser-Meyer-Olkin measure  of sampling adequacy** | 0.939 | | | | | |
| ^a^ Blanks represent absolute loading <0.32, the assumed threshold for salient loading.  Abbreviations: EQ-5D-5L: EuroQoL 5-Dimension 5-Level, ICECAP-A: ICEpop CAPability measure for Adults, ESS: Epworth Sleepiness Scale, FOSQ: 10-item Functional Outcomes of Sleep Questionnaire, PSQI: Pittsburgh Sleep Quality Index, n/a: not applicable. | | | | | | |

Table S8. Results of exploratory factor analysis excluding outlier observations showing factor loadings of the EQ-5D-5L, ICECAP-A and PSQI dimensions, and ESS and FOSQ-10 items.^a^

| **Variable** | **Factor 1** | **Factor 2** | **Factor 3** | **Factor 4** | **Factor 5** | **Uniqueness** |
| --- | --- | --- | --- | --- | --- | --- |
| **EQ-5D-5L Dimensions** |  |  |  |  |  |  |
| Mobility |  |  |  | 0.86 |  | 0.26 |
| Self-care |  |  |  | 0.78 |  | 0.16 |
| Usual activities |  |  |  | 0.76 |  | 0.21 |
| Pain/discomfort |  |  |  | 0.61 |  | 0.42 |
| Anxiety/depression |  |  | 0.47 |  |  | 0.55 |
| **ICECAP-A Dimensions** |  |  |  |  |  |  |
| Stability |  |  | 0.85 |  |  | 0.26 |
| Attachment |  |  | 0.77 |  |  | 0.43 |
| Autonomy |  |  | 0.40 | 0.45 |  | 0.47 |
| Achievement |  |  | 0.77 |  |  | 0.29 |
| Joy |  |  | 0.78 |  |  | 0.29 |
| **ESS Items** |  |  |  |  |  |  |
| Sitting and reading |  | 0.77 |  |  |  | 0.41 |
| Watching TV |  | 0.77 |  |  |  | 0.46 |
| Sitting, inactive |  | 0.78 |  |  |  | 0.27 |
| Car passenger |  | 0.73 |  |  |  | 0.38 |
| Lying down to rest |  | 0.62 |  |  |  | 0.60 |
| Sitting and talking | -0.37 | 0.57 |  |  |  | 0.23 |
| Sitting quietly |  | 0.72 |  |  |  | 0.31 |
| In a car | -0.44 | 0.53 |  |  |  | 0.24 |
| **FOSQ-10 Items** |  |  |  |  |  |  |
| Concentration | 0.74 |  |  |  |  | 0.29 |
| Remembering | 0.74 |  |  |  |  | 0.32 |
| Finishing a meal | 0.86 |  |  |  |  | 0.22 |
| Working on a hobby | 0.79 |  |  |  |  | 0.26 |
| Housework | 0.76 |  |  |  |  | 0.30 |
| Operating motor vehicle (short distance) | 0.92 |  |  |  |  | 0.17 |
| Operating a motor vehicle (long distance) | 0.76 |  |  |  |  | 0.37 |
| Difficulty completing tasks | 0.90 |  |  |  |  | 0.20 |
| Managing financial affairs | 0.92 |  |  |  |  | 0.22 |
| Performing work | 0.87 |  |  |  |  | 0.20 |
| **PSQI Components** |  |  |  |  |  |  |
| Sleep quality |  |  |  |  | 0.69 | 0.35 |
| Sleep latency |  |  |  |  | 0.55 | 0.56 |
| Sleep duration |  |  |  |  | 0.83 | 0.44 |
| Sleep efficiency |  |  |  |  | 0.81 | 0.47 |
| Sleep disturbances |  |  |  |  | 0.38 | 0.57 |
| Use of sleep medication |  |  |  |  |  | 0.77 |
| Daytime dysfunction | -0.52 |  |  |  |  | 0.42 |
| **Total variance explained by factor (%)** | 38.1 | 11.7 | 5.9 | 5.1 | 3.8 | n/a |
| **Determinant** | *p* < 0.001 | | | | | |
| **Bartlett’s test of sphericity** | *X*^2^ = 28151.710, *p* < 0.001 | | | | | |
| **Kaiser-Meyer-Olkin measure  of sampling adequacy** | 0.927 | | | | | |
| ^a^ Blanks represent absolute loading <0.32, the assumed threshold for salient loading.  Abbreviations: EQ-5D-5L: EuroQoL 5-Dimension 5-Level, ICECAP-A: ICEpop CAPability measure for Adults, ESS: Epworth Sleepiness Scale, FOSQ: 10-item Functional Outcomes of Sleep Questionnaire, PSQI: Pittsburgh Sleep Quality Index, n/a: not applicable. | | | | | | |
